# Implementation of Chernobyl optimization algorithm based feature selection approach to predict software defects

**DOI:** 10.12688/f1000research.150927.1

**Published:** 2024-07-29

**Authors:** Kunal Anand, Ajay Kumar Jena, Himansu Das

**Affiliations:** 1 School of Computer Engineering, Kalinga Institute of Industrial Technology, Bhubaneswar, Odisha, 751024, India

**Keywords:** Software Defect Prediction; Feature Selection; Wrapper approach; Chernobyl Disaster Optimizer, Optimization

## Abstract

**Background:**

Software defects can have catastrophic consequences. Therefore, fixing these defects is crucial for the evolution of software. Software Defect Prediction (SDP) enables developers to investigate unscramble faults in the inaugural parts of the software progression mechanism. However, SDP faces many challenges, including the high magnitude of attributes in the datasets, which can degrade the prognostic performance of a defect forecasting model. Feature selection (FS), a compelling instrument for overcoming high dimensionality, selects only the relevant and best features while carefully discarding others. Over the years, several meta-heuristic algorithms such as the Genetic Algorithm (GA), Particle Swarm Optimization (PSO), Differential Evolution (DE), and Ant Colony Optimization (ACO) have been used to develop defect prediction models. However, these models suffer from several drawbacks, such as high cost, local optima trap, lower convergence rate, and higher parameter tuning. To overcome the above shortcomings, this study aims to develop an innovative FS technique, namely, the Chernobyl Optimization Algorithm (FSCOA), to unwrap the most informative features that can produce a precise prediction model while minimizing errors.

**Methods:**

The proposed FSCOA approach mimicked the process of nuclear radiation while attacking humans after an explosion. The proposed FSCOA approach was combined with four widely used classifiers, namely Decision Tree (DT), K-nearest neighbor (KNN), Naive Bayes (NB), and Quantitative Discriminant Analysis (QDA), to determine the finest attributes from the SDP datasets. Furthermore, the accuracy of the recommended FSCOA method is correlated with existing FS techniques, such as FSDE, FSPSO, FSACO, and FSGA. The statistical merit of the proposed measure was verified using Friedman and Holm tests.

**Results:**

The experimental findings showed that the proposed FSCOA approach yielded the best accuracy in most cases and achieved an average rank of 1.75, followed by the other studied FS approaches. Furthermore, the Holm test showed that the p-value was lower than or equivalent to the value of α/(A-i), except for the FSCOA and FSGA and FSCOA and FSACO models.

**Conclusion:**

The experimental findings showed that the prospective FSCOA procedure eclipsed alternative FS techniques with higher accuracy in almost all cases while selecting optimal features.

## Introduction

In today’s scenario, humankind needs good-quality and reliable software to help them perform their daily tasks without spending more time and effort. Owing to this immense call for exceptional and dependable software, conducting a rigorous investigation of software under development is crucial. However, the complexity of software increases every passing day, making the overall software development work very challenging. ^1^^,^^2^ A fault in software can significantly damage its quality and reliability, leading to more frequent maintenance activities. This can result in higher operational costs for the software, ultimately leading to user dissatisfaction. A software fault can be characterized as the disparity between the actual and predicted behaviors of the software. Software testing empowers developers to identify and rebuild faults. However, conventional testing approaches are costly and time consuming. Hence, it is imperative to detect faults in a software module during the early stages of evolution. ^3^

Software Defect Prediction (SDP) enables developers to expose deficiencies in software components in the early stages of development by employing data analysis and machine learning (ML) approaches. An effective SDP mechanism can lead to the systematic and profitable advancement of high-quality and reliable software products without defects. ^4^ Researchers have suggested several ML-based SDP approaches ^5^^–^^8^ for effectively predicting defects. These methods analyze past data from different stages of development, such as testing data and debugging records, to derive any pattern or trend that can detect any potential defects. The most widely employed ML methods in SDP are DT, ^9^ SVM, ^10^ neural networks, ^11^ logistic regression, ^12^ and NB. ^13^ However, these approaches suffer from many challenges including high dimensionality being one of them.

Feature Selection (FS) ^14^ is a potent mechanism that can be employed to overcome the issue of high dimensionality. FS allows developers to select only relevant features and carefully discard insignificant features. In SDP, FS is a vital step that allows developers to choose the best set of features that can significantly enhance the predictive accuracy of a defect prediction model. The application of FS approaches is essential when dealing with datasets with high dimensionality. Several FS approaches have been implemented in the SDP. FS techniques are broadly classified into three categories: filter techniques, ^15^ wrapper techniques, ^16^ and embedded techniques. ^17^^,^^18^ Filter-based FS techniques are autonomous for any training strategy, and apply statistical properties to identify the best traits. Nonetheless, wrapper-based FS procedures select the best characteristics based on the classification accuracy of the prediction model. Embedded-based FS techniques combine feature selection with model training. Existing literature shows that researchers have mainly applied evolution-based algorithms ^19^ and swarm-based algorithms ^20^ for FS purposes.

This exploration aims to boost the classification truthfulness of a defect-foretelling model while minimizing errors. For this purpose, this study uses some of the widely used meta-heuristic algorithms, namely the Genetic Algorithm (GA), ^21^ Particle Swarm Optimization (PSO), ^22^^,^^23^ Differential Evolution (DE), ^24^ and Ant Colony Optimization (ACO). ^25^ Although GA has been a proven FS approach, ^26^ it is costly because it computes the optimal features using genetic techniques such as selection, crossover, and mutation over a set of generations. The PSO-based FS approach ^27^ aims to find the optimal traits by emulating the fragment’s movement while probing a search arena with several dimensions. The algorithm adjusts the location and velocity of the fragment by considering singular and group knowledge. However, the PSO-based FS approach sometimes results in a local optima trap in addition to a lower convergence rate. DE-based FS techniques ^28^ compute optimal characteristics by employing operators such as mutation, crossover, and selection on a population of potential solutions over several iterations. However, these approaches require several parameter tunings, making them a tedious choice among researchers. ACO-based FS methods ^29^ determine the excellent characteristics of a search space by implementing the foraging behavior of ants. However, these methods suffer from a slow convergence speed and low accuracy, especially in large datasets.

The limitations of the aforementioned FS approaches motivated us to propose a novel FS approach (FSCOA) inspired by the Chernobyl Disaster Optimizer (CDO). ^30^ The primary objective of the proposed FSCOA approach is to unwrap the most informative features to produce a precise prediction model. It mimics the process of nuclear radiation, which involves the propagation of alpha, beta, and gamma fragments while attacking humans after an explosion. These radiations fly at a very high speed from a high-pressure point (the point of explosion) to a low-pressure point (the standing of the individual place). The proposed algorithm comprises an initial population of the candidate solutions. Furthermore, it computes the gradient descent factor (GDF) for alpha, beta, and gamma fragments when they attack humans. Finally, the optimal solution was achieved by calculating the average of the GDF values over several iterations. The proposed algorithm has advantages such as its ability to deal with convoluted, high-magnitude datasets that are not grounded in local optima, which can be an issue in alternate FS procedures. The primary contributions of this study are as follows.

(i) To develop a novel FSCOA approach by applying the CDO, a metaheuristic algorithm
(ii) To evaluate the performance of the proposed FSCOA-based fault forecasting model on four different classification algorithms, NB, QDA, DT, and KNN, 12 benchmark NASA software defect datasets were used.
(iii) To correlate the performance of the proposed FSCOA approach with several FS approaches such as FSGA, FSPSO, FSDE, and FSACO.
(iv) To validate the statistical implications of the proposed FSCOA approach using Friedman and Holm tests.

The experimental outcome shows that the proposed FSCOA was better than the other FS approaches examined in most situations and then became the best-performing FS technique for designating the best array of features. The remainder of this paper is organized as follows. Section 2 discusses the existing literature on FS approaches. Segment 3 elaborates on the proposed FSCOA approach and the detailed methodology used in this study. Segment 4 presents the empirical findings and interpretations. Segment 5 outlines the statistical analysis. Finally, Segment 6 presents the conclusions and scope for prospective work.

## Related works

Defect prediction in software modules plays a critical role in creating high-quality and reliable software. SDP permits developers to detect and debug defects in software modules during the prior stage of the software advancement process. Unfortunately, conventional SDP processes face several threats, one of which is the curse of dimensionality. The curse of dimensionality indicates the presence of a large number of attributes in a dataset. Many of these attributes do not make any compelling knowledge, and hence, are treated as noise. Feature selection is a potent tool to tackle the challenge of the curse of dimensionality. FS allows developers to establish the best possible set of traits that can enhance the predictive accuracy of the model by discarding irrelevant traits. However, it is imperative to observe that conventional FS procedures are not only expensive but also time-consuming. ^31^ Recently, the application of ML to SDP has gained considerable traction. Several ML-based SDP approaches have been proposed. This section describes some of these studies as follows.

Das et al. ^32^ proposed a novel FS technique called FSGJO based on the Golden Jackal Optimization (GJO) algorithm. The proposed FSGJO technique was employed on four classifiers, namely, KNN, DT, NB, and QDA, using 12 SDP datasets taken from the PROMISE repository. The authors compared the efficacy of the recommended FSGJO technique with alternative FS techniques, namely, FSDE, FSPSO, FSACO, and FSGA. Based on their experimental findings, the authors observed that the proposed FSGJO technique enhanced the prognostic performance of the model. It was also noted that the prospective FSGJO method was exceptional compared to other studied FS techniques in selecting the optimal set of characteristics. However, the authors mentioned that the proposed FSGJO technique needs its parameters to be tuned.

Khalid et al. ^33^ inspected numerous existing ML methods and optimized ML procedures on three publicly accessible NASA datasets. The authors applied PSO and ensemble approaches in their work and scrutinized the results. The experimental findings revealed that the SVM and optimized SVM outperformed the other models in terms of accuracy. However, this study was conducted using a limited number of datasets. Again, the experimental findings cannot be generalized owing to the lack of additional optimization algorithms.

Kumar and Das ^34^ enforced GA to supervise learning classifiers such as KNN, DT, and NB. Twelve NASA datasets from the PROMISE archive were used. Using accuracy and failure rate as performance metrics, the performance of the proposed model was assessed. Based on their experimental results, the authors asserted that the suggested FSGA technique improved the behavior of the defect forecast model correlated with the scenario in which the FS was not made. However, in this study, the FS approach used only the GA. The effects of alternative optimization methodologies were not investigated.

Thirumoorthy et al. ^35^ suggested a hybrid SDP method based on the TOPSIS and hrbrid Rao algorithms (THRO) to uncover the finest set of traits. The authors used three benchmark NASA SDP datasets to implement their proposed THRO-based FS algorithm on SVM and NB classifiers. The impact of the proposed algorithm was assessed using six metaheuristic FS techniques. The authors noted that the proposed THRO-based FS algorithm enhanced the classification performance of the model and outperformed other studied FS approaches. However, they also noted that this enhanced performance of the proposed method came at the price of increased computational cost.

Batool et al. ^36^ offered a comprehensive and well-organized analysis of the extant literature. They looked at numerous pertinent published publications that employed DM, ML, and DL, among other techniques, for fault prediction. The endeavor was motivated by the need to find answers to research problems stated in the evaluation that might not have been addressed in the works evaluated or called for a different viewpoint. The authors claim that DM and ML techniques, such as DT, NB, SVM, NN, ET, and EA, are frequently employed by SDP. Although they are used less frequently, DL approaches such as CNN, MLP, LSTM, and DNN have also been used by researchers to predict software errors. The authors emphasized the need for larger datasets and the importance of concentrating on using the same methods with combinations of different datasets.

An SDP architecture based on stacked stacking and heterogeneous FS was proposed by Chen et al. ^37^ The two main objectives of this study were to increase SDP accuracy and optimize software testing resource allocation. The method is divided into three steps: feature selection, model creation with a nested-stacking classifier, and evaluation of the predictive behavior of the model. For the experiments, two datasets were used: Kamei and PROMISE. The investigation included both within-project and large-scale cross-project defect prediction. The model’s behavior was illustrated using the AUC and F1-score evaluation metrics. The initial results showed that for the two sets of software failure datasets, the proposed framework performed better in terms of classification than the baseline models. However, the authors pointed out that nested-stacking is not very effective and that the optimal combination of the baseline model was determined via difficult experiments.

Arora and Kaur ^38^ suggested a method that used FS on both origin and destination datasets to assemble a heterogeneous fault prediction (HFP) model to develop an effective forecasting model utilizing supervised training approaches. The writers completed the FS in two phases. They began by selecting features based on their importance. They removed the shared features from the datasets in the next step. An integrated approach was used to select the best characteristics. RFI was used for the FS. According to the suggestion made by Gao et al., ^39^ the authors selected the top 15% of attributes throughout the FS phase. The proposed framework was applied to two open-source projects, MySQL and Linux, for the supervised ML classifiers, SVM, NB, RF, AdaBoost, DT, and LR. The behavior of the planned model was graded using the Area under the ROC curve (AUC). The authors concluded that the most accurate logistic regression fault prediction is based on the recommended approach. The AUC data demonstrated that the suggested technique accomplished better than the existing Cross Project Fault Prediction (CPFP). However, in this study, other commonly used performance criteria such as accuracy, precision, and recall were not employed to grade the impact of the proposed approach. Once again, only supervised learning algorithms were used in the study, and no optimization algorithms were used.

Anand et al. ^40^ conducted a correlative performance assessment of various FS techniques utilized in SDP. Chi-Square (CS), Correlation Coefficient (CC), Fisher’s Score, Information Gain (IG), Mean Absolute Difference (MAD), and Variance Threshold (VT) are among the filter-based FS approaches used in this investigation. Wrapper-based FS strategies also encourage the use of Backward Feature Elimination (BFE), Exhaustive Feature Elimination (EFE), Forward Feature Elimination (FFE), and Recursive Feature Elimination (RFE) methodologies. RFI and LASSO Regularization are among the embedded FS techniques utilized in this study. The recommended model uses six publicly accessible benchmark NASA datasets for the NB, SVM, DT, and KNN classifiers. The authors used the F1-score, recall, accuracy, and precision as performance evaluation criteria. The authors’ experimental results showed that Fisher’s score behaved more precisely than other FS techniques. However, compared to the no-FS situation, it was found that all FS strategies enhanced the model’s behavior. A drawback of this study is that it neglected to examine the impact of optimization strategies on the FS.

The dynamic re-ranking approach-based WFS technique was introduced by Balogun et al. ^41^ in response to the exorbitant processing expenses of wrapper-based FS (WFS) methods. The recommended technique was constructed using 25 public domain datasets that were extracted from the NASA, AEEEM, PROMISE, and ReLink archives using classifiers such as DT and NB. The findings of the experiment illustrated that the recommended method reduced computing time and enhanced model performance when executing FS. The suggested method was performed using both the FFS and WFS techniques, which is a disadvantage. FFS has variable performance across datasets and classifiers, whereas WFS suffers from stagnation of local optima and high computing costs. Once more, only two supervised classifiers were examined in this work: SVM and K-NN, two more well-known classifiers, were not examined.

Balogun et al. ^42^ proposed an inventive hybrid multifilter wrapper FS arrangement based on rank aggregation to select critical features to address the aforementioned shortcomings. The recommended course of action was implemented in two steps. In the first lap, a multifilter FS mechanism based on rank aggregation was used, which combined the separate rank lists from multifilter methods to build an original, dependable, and non-disjoint rank list. This resolves the filter rank choice issue. In the second lap, the upgraded wrapper FS approach, which was predicated on dynamic re-ranking, was used once more to preprocess the accumulated ranked attributes. The competence of the recommended method is illustrated using NB and DT classifiers on benchmark software fault datasets. The tests used accuracy, area under the curve (AUC), and F-measure values as evaluation criteria. The authors used their findings to concentrate on the issues of filter rank choice and local optima stagnation in HFS, demonstrating the suggested method’s ingenuity in selecting the best characteristics while enduring or boosting the impact of the forecasting models. They concluded that applying the recommended technique significantly improves the behavior of the model. However, the model was limited to only two classifiers to achieve satisfactory results. Consequently, the potential of extrapolating the results to alternative classifiers has not been explored.

Alsghaier and Akour ^43^ presented an SDP model by fusing the GA, SVM, and PSO. Three stages were implemented: GA-SVM for GA integration, PSO-SVM for PSO integration, and GAPSO_SVM for the reciprocal iteration-based integration of GA-SVM and PSO-SVM. During the experimentation phase, 24 benchmark SDP datasets (12 NASA MDP and 12 open-source Java applications) were subjected to the proposed model using the SVM classifier. To validate the theoretical model, experiments were conducted using the WEKA Tool and MATLAB 2015. The impact of the developed approach was assessed using evaluation metrics, such as accuracy, recall, precision, F-measure, specificity, error rate, and standard deviation. The results of the experiment showed that combining the GA with SVM and PSO had a beneficial effect on the model and enhanced its performance when applied to both small- and large-scale datasets. However, the precision metric was insufficient to appraise the suggested procedures.

Alsghaier and Akour ^44^ built on their earlier work ^43^ by combining GA, SVM, and Whale Optimization Algorithm (WOA) to forecast defects. The remainder of the experimental configuration remained the same as in a previous study. ^43^ Through experimental data, the researchers discovered that the behavior of the defect forecast model was improved for both large-scale and small-scale datasets when the GA was integrated with SVM and WOA. For the datasets under study, WA-SVM performed more accurately than GAWA-SVM, and GAWA-SVM produced the worst outcomes. Again, the proposed method outperformed SVM for the NASA MDP and open-source Java projects in terms of SD scores. This illustrates how combining SVM with optimization techniques enhances the prediction performance. The NASA, GA-SVM, and GAWA-SVM datasets produced the best outcome in terms of specificity. This proved that the GA-SVM and GAWA-SVM procedures are appropriate for software defect predictions when enforced on an enormous dataset.

Balogun et al. ^45^ used NASA datasets from the PROMISE archives to thoroughly evaluate the FSS algorithms on NB, DT, LR, and KNN. Their findings imply that the studied FS techniques enhanced the performance of the system. Information Gain, one of the FFR techniques, demonstrated the best results. Consistency Feature Subset Selection (CFSS), which is based on the Best First Search in FSS methods, has the greatest impact on forecasting models. However, there were variations in the performances of the classifiers’ and datasets.’ Scientists have also found that models constructed using FFR-based techniques are more stable than those constructed using FSS-based approaches. This study focused only on FFS procedures, and the effects of the WFS techniques were not investigated in detail.

All of the previously stated FS approaches, whether supervised or unsupervised, have disadvantages that significantly impact the model’s performance, including (i) high cost, (ii) entrapment in local optima, (iii) low convergence rate, and (iv) fine-tuning of excessively many parameters. The primary drawback of the previously stated FS techniques is the need to modify the regulating parameters accurately while choosing ideal characteristics. These shortcomings motivated us to propose a novel FS technique (FSCOA) that draws inspiration from the Chernobyl Disaster Optimizer (CDO). The 1986 nuclear reactor core outburst in Chernobyl served as an impetus for the development of the CDO meta-heuristic algorithm. The process of nuclear radiation, in which alpha, beta, and gamma fragments propagate and damage humans following an explosion, is replicated by CDO. From the high-pressure point (explosion site) to the low-pressure point (individual standing standing), the above-mentioned radiations travel at an extremely rapid speed. The algorithm comprises an initial population of potential solutions. Moreover, it calculates the alpha, beta, and gamma fragment gradient descent factors (GDF) during human attacks. Determining the average of these GDF values over a number of iterations yields the best result.

### Feature selection using Chernobyl Optimization Algorithm

**Figure 1.**
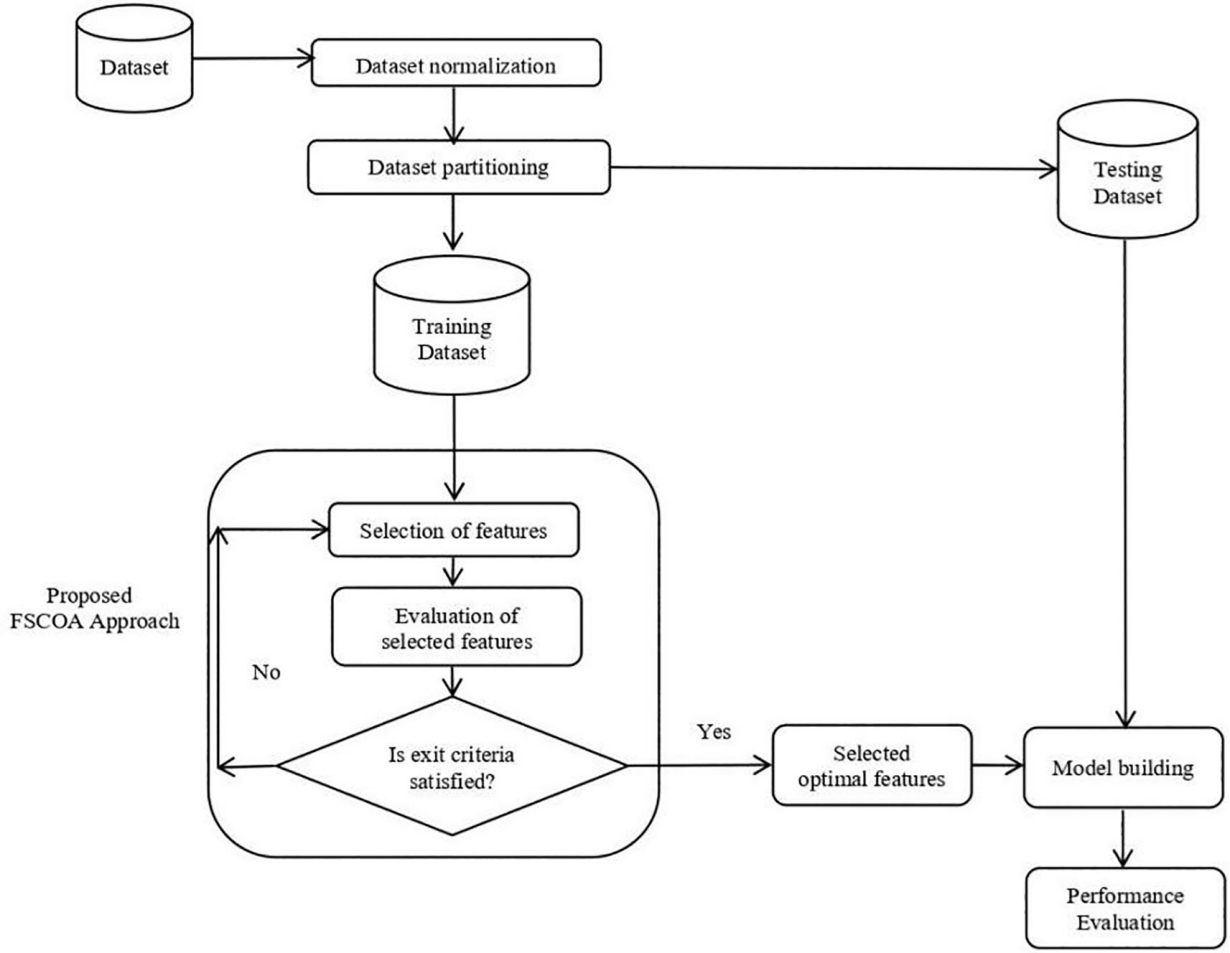
Blueprint of the suggested FSCOA methodology.

Feature selection determines the crucial attributes that have the greatest impact on the desired variable, which helps increase machine learning model accuracy, reduce computing costs, and reduce the risk of overfitting. The mechanism of selecting the best features consists of three steps: (1) creating a set of subgroups of attributes; (2) assessing and comparing the adequacy of these subgroups to determine which subgroup is the best or until the abort standards are met; and (3) computing the outcome using only the best features. It is difficult to determine which parts to classify. The 1986 Chernobyl nuclear reactor catastrophe ^46^ is recognized as one of the lowest nuclear disasters in the modern human past, in terms of both cost and casualties. Inspired by the Chernobyl nuclear reactor core eruption, the Chernobyl Disaster Optimization (CDO) ^30^ is a meta-heuristic optimization technique. In order to choose the most appropriate subset of characteristics for classification, a novel FS approach using Chernobyl Optimization Algorithm **(**FSCOA) is therefore proposed in order to address the aforementioned problem. Figure 1 ^53^ shows the blueprint for the proposed FSCOA method.

First, the selection of the relevant SDP datasets is crucial. Following the selection of the datasets, an in-depth examination of the datasets was carried out to determine any missing, inconsistent, or categorical data. It became apparent that there were no missing data in the datasets. Nevertheless, a few datasets contained categorical data. The data were categorized prior to the generation of the numbers. Furthermore, the original feature value, which originally ranged from 0 to 1, was normalized. Subsequently, an 80:20 split between the training and testing datasets was created for each normalized dataset. To develop and investigate the model, the two preeminent criteria are the population size and maximum number of iterations. Higher values will improve the performance of the model, but they also lengthen the computation time. In this study, the population size and maximum tally of the iterations were set to 30 and 200, respectively. By applying the recommended FSCOA methodology, four supervised learning classifiers (DT, KNN, NB, and QDA) were used to construct the model using the optimal features that were chosen. The best predictive classifier was then determined by comparing the accuracy of the proposed FSCOA approach with that of the other FS models under study.

**Figure 2.**
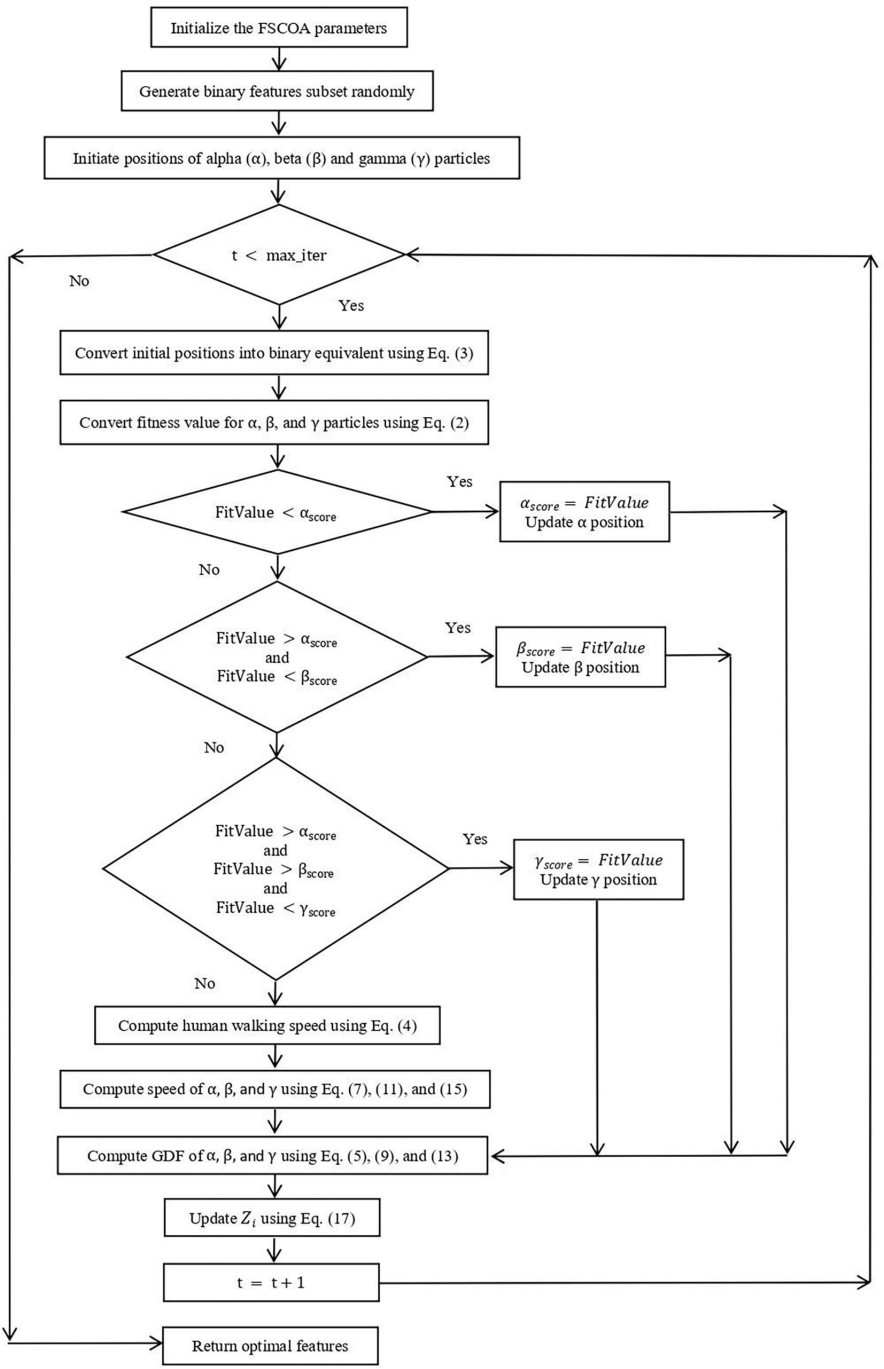
Flow-diagram of recommended FSCOA approach.

This study suggests a novel FSCOA technique to select the first-rate subgroup of attributes for categorization. The primary intent of the suggested technique is to identify the best attribute combination that will lower the fitness of the model. Figure 2 ^53^ shows a complete flow diagram of the proposed FSCOA technique.

Initializing the criteria, such as population size (M) , problem dimension (F) , lower bound (LowBound) , and upper bound (UppBound) , is the first step of the procedure. Subsequently, a random binary population of M fragments with dimension F, where $Z=\left[Z_{1},Z_{2},Z_{3},\ldots ,Z_{M}\right]$ is the total number of features. $Z_{i}=\left[Z_{1},Z_{2},Z_{3},\ldots ,Z_{M}\right]$ is the $i^{\mathrm{th}}$ fragment location in the F dimension feature space, where i=1,2,3,…,M is the specimen proportion, and $Z_{i,f}$ is the $i^{\mathrm{th}}$ fragment standing of the $f^{\mathrm{th}}$ trait of the population. Many classification techniques, including DT, KNN, NB, and QDA for fitness (error) computation, have been considered for the examination of randomly selected characteristics. The FS algorithm aims to select the best subset of ideal features that may reduce the fitness of the learning algorithm. The error $\left({\mathrm{Err}}_{i}^{t}\right)$ is estimated as the disparity between the estimated outcome $\left({\mathrm{EO}}_{i}^{t}\right)$ and actual outcome $\left({\mathrm{AO}}_{i}^{t}\right)$ . Eq. (1) can be used to describe this phenomenon.

$${\mathrm{Err}}_{i}^{t}={\mathrm{AO}}_{i}^{t}-{\mathrm{EO}}_{i}^{t}$$ (1)

By distributing the aggregate of the total errors by the entire count of instances in the testing data, the fitness ( ${\text{FitValue}}_{i}^{t}$ ) of the learning algorithm was calculated. This is characterized by Eq. (2).

$${\text{FitValue}}_{i}^{t}=\frac{\sum_{i=1}^{p}{\mathrm{Err}}_{i}^{t}}{p}$$ (2)

Here, i=1,2,…..,p and p represent the tally of the instances in the trial data, t represents the current iteration.

The transfer function depicted in Eq. (3) was employed to transform the initial fragment standings into a binary equivalent.

$$\mathrm{TF}=\frac{1}{1+\left(\exp^{\left(-10\times \left(Z_{i,f}-0.5\right)\right)}\right)}$$ (3)

The proposed FSCOA approach employs the CDO algorithm to determine the optimal features for a given dataset. In CDO, different types of emissions are released from the nuclei as a result of radioactivity caused by nuclear instability. The most prevalent types of these emissions are alpha, beta, and gamma fragments. These fragments, which are very dangerous to people, fly from a high-pressure point (the point of explosion) to a low-pressure point (the standing of individual standing). When a human is attached to a CDO following a nuclear explosion, it simulates the effects of radioactive decay. The primary processes of nuclear explosion and human attachment require the use of gamma, beta, and alpha fragments. Humans were most likely to be on foot when they were attacked. Human walking speed can be enhanced, and it can be estimated to be between 0 and 3 miles per hour. ^49^ Based on this, Eq. (4) can be used to model linear reduction at this speed.

$${\text{WalkSpeed}}_{\text{human}}=3-t*\left(\left(3\right)/\text{max}\_\text{iter}\right)$$ (4)

Alpha fragment

The gradient descent factor $\left({\mathrm{GDF}}_{\alpha }\right)$ of the alpha fragment while threatening humans can be computed using Eq. (5).

$${\mathrm{GDF}}_{\alpha }=0.25\times \left({\mathrm{POS}}_{\alpha }\;\left(t\right)-{\text{PROP}}_{\alpha }\times D_{\alpha }\right)$$ (5)

Here, ${\mathrm{POS}}_{\alpha }\;\left(t\right)$ is the prevailing standing of alpha fragments, ${\text{PROP}}_{\alpha }$ represents the dispersion of alpha fragments and can be calculated using Eq. (6); $D_{\alpha }$ is the discrepancy between the individual standing and standing of alpha fragments, which can be determined using Eq. (8).

$${\text{PROP}}_{\alpha }=\frac{\pi \times \mathrm{rad}\times \mathrm{rad}}{0.25\times {\text{Speed}}_{\alpha }}-\left({\text{WalkSpeed}}_{\text{human}}\times \text{rand}\;\left(\right)\right)$$ (6)

Here, rad is a random value between 0 and 1, ${\text{Speed}}_{\alpha }$ is the speed of alpha fragments that can be in the range of 1–16,000 kmps. This can be normalized using Eq. (7)

$${\text{Speed}}_{\alpha }=\text{log}\;\left(\text{rand}\left(1:16000\right)\right)$$ (7)

$$D_{\alpha }=\left|{\text{Area}}_{\alpha }\times {\mathrm{POS}}_{\alpha }\;\left(t\right)-{\mathrm{Avg}}_{T}\;\left(t\right)\right|$$ (8)

Here, ${\text{Area}}_{\gamma }$ is the propagation area of gamma fragments that can be calculated as π∗rad∗rad where rad is a random value between 0 and 1, ${\mathrm{Avg}}_{T}$ is the average of the total standings that can be determined using Eq. (17).

***Beta fragment***

Eq. (9) can be used to determine the gradient descent factor $\left({\mathrm{GDF}}_{\beta }\right)$ of a beta fragment assaulting a human.

$${\mathrm{GDF}}_{\beta }=0.5\times \left({\mathrm{POS}}_{\beta }\;\left(t\right)-{\text{PROP}}_{\beta }\times D_{\beta }\right)$$ (9)

Here, ${\mathrm{POS}}_{\beta }\;\left(t\right)$ is the current standing of beta fragments; ${\text{PROP}}_{\beta }$ represents the propagation of beta fragments and can be calculated using Eq. (10); $D_{\beta }$ is the discrepancy between the human standing and the beta fragment’s standing, which can be determined using Eq. (12).

$${\text{PROP}}_{\beta }=\frac{\pi \times \mathrm{rad}\times \mathrm{rad}}{0.5\times {\text{Speed}}_{\beta }}-\left({\text{WalkSpeed}}_{\text{human}}\times \text{rand}\;\left(\right)\right)$$ (10)

Here, rad is a random value between 0 and 1, ${\text{Speed}}_{\beta }$ is the speed of beta fragments that can be in the range of 1–270,000 kmps. This can be normalized using Eq. (11)

$${\text{Speed}}_{\beta }=\text{log}\;\left(\text{rand}\left(1:270000\right)\right)$$ (11)

$$D_{\beta }=\left|{\text{Area}}_{\beta }\times {\mathrm{POS}}_{\beta }\;\left(t\right)-{\mathrm{Avg}}_{T}\;\left(t\right)\right|$$ (12)

Here, ${\text{Area}}_{\beta }$ is the propagation area of the beta fragment and can be calculated as π∗rad∗rad where rad is a random value between 0 and 1. ${\mathrm{Avg}}_{T}$ is the average of the total standings that can be computed using Eq. (17).

***Gamma fragments***

The gradient descent factor $\left({\mathrm{GDF}}_{\gamma }\right)$ of the gamma fragment while making an assault on humans can be computed using Eq. (13).

$${\mathrm{GDF}}_{\gamma }=\left({\mathrm{POS}}_{\gamma }\;\left(t\right)-{\text{PROP}}_{\gamma }\times D_{\gamma }\right)$$ (13)

Here, ${\mathrm{POS}}_{\gamma }\;\left(t\right)$ is the prevailing standing of gamma fragments, ${\text{PROP}}_{\gamma }$ represents the dispersion of gamma fragments and can be calculated using Eq. (14); $D_{\gamma }$ is the discrepancy between the standing of the human and the standing of gamma fragments, which can be determined using Eq. (16).

$${\text{PROP}}_{\gamma }=\frac{\pi \times \mathrm{rad}\times \mathrm{rad}}{{\text{Speed}}_{\gamma }}-\left({\text{WalkSpeed}}_{\text{human}}\times \text{rand}\;\left(\right)\right)$$ (14)

Here, rad is a random value between 0 and 1, ${\text{Speed}}_{\gamma }$ is the speed of the gamma fragment in the range of 1 to 300,000 kmps. This can be normalized using Eq. (15)

$${\text{Speed}}_{\gamma }=\text{log}\;\left(\text{rand}\left(1:300000\right)\right)$$ (15)

$$D_{\gamma }=\left|{\text{Area}}_{\gamma }\times {\mathrm{POS}}_{\gamma }\;\left(t\right)-{\mathrm{Avg}}_{T}\;\left(t\right)\right|$$ (16)

Here, ${\text{Area}}_{\gamma }$ is the propagation area of gamma fragments that can be calculated as π∗rad∗rad where rad is a random value between 0 and 1, ${\mathrm{Avg}}_{T}$ is the average of the total standings that can be determined using Eq. (17).

$${\mathrm{Avg}}_{T}=\left(\frac{{\mathrm{GDF}}_{\alpha }+{\mathrm{GDF}}_{\beta }+{\mathrm{GDF}}_{\gamma }}{3}\right)$$ (17)

Finally, Algorithm 1 provides a summary of the entire proposed FSCOA process.

Algorithm 1. **Proposed FSCOA approach**

1. Initialize Populace Size (M) , Dimension (F) , Lower Bound (LowBound) , UpperBound (UppBound) , Maximum Iteration (max_iter)

2. Generate the binary feature subset $Z_{i}$ randomly
3. Initialize the alpha ( ${\mathrm{POS}}_{\alpha }$ ), beta ( ${\mathrm{POS}}_{\beta }$ ), and gamma ( ${\mathrm{POS}}_{\gamma }$ ) standings
4. while (t<max_iter) do{
5.   for i=1 : M do
6.    for j=1to F do
7.     The values of the initial standing of the fragments are converted into their corresponding binary values using Eq. (3).
8.     Compute the fitness value (FitValue) for the alpha, beta, and gamma fragments using Eq. (2)
9.     if $\left(\text{FitValue}<\propto_{\text{score}}\right)$

10.
$\;\;\;\;\;\;\;\;\;\;\;\propto_{\text{Score}}=\text{FitValue}$

11.        Update ${\mathrm{POS}}_{\alpha }$

12.     endif
13.     if $\left(\text{FitValue}>\propto_{\text{score}}\right)\;\text{and}\;\left(\text{FitValue}<\beta_{\text{score}}\right)$

14.
$\;\;\;\;\;\;\;\;\;\;\;\beta_{\text{Score}}=\text{FitValue}$

15.        Update ${\mathrm{POS}}_{\beta }$

16.     endif
17.     if $\left(\text{FitValue}>\propto_{\text{score}}\right)\;\text{and}\;\left(\text{FitValue}>\beta_{\text{score}}\right)\;\text{and}\;\left(\text{FitValue}<\gamma_{\text{score}}\right)$

18.
$\;\;\;\;\;\;\;\;\;\;\;\gamma_{\text{Score}}=\text{FitValue}$

19.        Update ${\mathrm{POS}}_{\gamma }$

20.     endif
21.    end for
22.   end for
23. Compute human walking speed $\left({\text{WalkSpeed}}_{\text{human}}\right)$ using Eq. (4)
24. Compute the speed of alpha $\left({\text{Speed}}_{\alpha }\right)$ , beta $\left({\text{Speed}}_{\beta }\right)$ , and gamma $\left({\text{Speed}}_{\gamma }\right)$ fragments using Eq. (7), Eq. (11), and Eq. (15), respectively.
25. for i=1 : M do
26.    for j=1 : F do
27.     Determine ${\mathrm{GDF}}_{\alpha }$ using Eq. (5)
28.     Determine ${\mathrm{GDF}}_{\beta }$ using Eq. (9)
29.     Determine ${\mathrm{GDF}}_{\gamma }$ using Eq. (13)
30.     Update $Z_{i}$ using average of total standings using Eq. (17)
31.    end for
32. end for
33.
t=t+1

34. } //end of while loop
35. Return finest solution, $Z_{i}$

36. end procedure

### Result analysis

This section deliberates on the empirical findings of this research. The persuasiveness of the proposed FSCOA approach was graded by employing 12 publicly benchmarked NASA software defect datasets extracted from the PROMISE archive. ^48^ KC1, KC3, CM1, JM1, MC1, MC2, MW1, PC1, PC2, PC3, PC4, and PC5 were the datasets. First, an in-depth examination of the datasets was performed to identify missing, inconsistent, and categorical data. It became apparent that there were no missing data in the datasets. Nevertheless, a few datasets contained categorical data. The data were categorized prior to the generation of the numbers. Again, we noticed that the datasets comprised of continuous data. The datasets were altered using the min–max normalization method ^49^ with the goal of overcoming this problem. The original feature value, which originally ranged from zero to one, was transformed using this technique. Subsequently, an 80:20 split between the training and testing datasets was created for each normalized dataset. Extensive information regarding the datasets enforced in this exploration is shown in Table 1.

**Table 1.** Specifics of the enforced NASA datasets.

Datasets	No. of instances	No. of features	Non-susceptible classes (SC)	Susceptible classes (SC)	Susceptible (%)
PC1	705	38	644	61	8.7
PC2	745	37	729	16	2.1
PC3	1077	38	943	134	12.4
PC4	1287	38	1110	177	13.8
PC5	1711	39	1240	471	27.5
CM1	327	38	285	42	12.8
JM1	7782	22	6110	1672	21.5
KC1	1183	22	869	314	26.5
KC3	194	40	158	36	18.5
MC1	1988	39	1942	46	2.3
MC2	125	40	81	44	35.2
MW1	253	38	226	27	10.6

The configuration of the computer on which the experiments were administered was as follows: Intel Core i5-6200 CPU with clock rate 2.40 GHz and 8 GB RAM. The aforementioned techniques were employed in a Python 3 environment using the Jupyter notebook. First, the input dataset was uploaded using Pandas. The datasets were altered using the min-max normalization method. ^49^ Using train_test_split from sklearn.model_selection, the dataset was partitioned into training and testing datasets at a ratio of 80:20. Populace size and the highest number of iterations were the two major criteria for developing and validating the model. The model will provide superior outcomes with higher values, but it will also increase computing time. In this investigation, the population size and highest number of iterations were set to 30 and 200, respectively. Four supervised learning classifiers, DT, KNN, NB, and QDA, were used to evaluate five FS approaches: FSDE, FSPSO, FSGA, FSACO, and the suggested FSCOA. The fitness error plots for the suggested FSCOA approach and other FS strategies utilizing the examined classifiers, DT, KNN, NB, and QDA, were obtained using matplotlib.pyplot. In this study, accuracy, a frequently applied performance indicator metric, was used for assessment purposes. Accuracy can be expressed as a simple proportion of the total instances of instances that were correctly classified. Eq. (18) is used to calculate it from the confusion matrix, as follows:

$$\text{Accuracy}=\frac{\mathrm{TP}+\mathrm{TN}}{\mathrm{TP}+\mathrm{TN}+\mathrm{FP}+\mathrm{FN}}$$ (18)

Here, TP , TN , FP , and FN represent true positives, true negatives, false positives, and false negatives, respectively.

The performance of the recommended FSCOA algorithm is evaluated against several FS procedures such as FSDE, FSPSO, FSGA, and FSACO in terms of classification accuracy and the count of selected attributes on 12 datasets studied in this research work. Because of the stochastic character of the previously mentioned techniques, we carried out ten runs of the trials to ensure that the performance of each procedure remained persistent, with an initial random population. The median accuracy of the proposed FSCOA, along with the other studied FS approaches, is listed in Table 2. ^54^

**Table 2.** Accuracy percentage and number of features selected by four classifiers for twelve datasets.

Sl. No.	Datasets	FS Algorithms/Classifiers	KNN	DT	NB	QDA	Attributes selected
1	KC1	Without FS	69.62	72.15	74.26	74.26	22
		FSDE	76.46	77.09	77.22	78.1	8.2
		FSPSO	74.64	73.12	76.12	76.27	8.3
		FSGA	76.47	76.03	77.22	77.93	9.4
		FSACO	77.69	76.85	77.47	77.85	4.5
		FSCOA	77.13	75.32	77.34	78.27	8.2
2	KC3	Without FS	74.36	76.92	66.67	76.92	40
		FSDE	80	90	76.92	86.92	17.7
		FSPSO	76.15	81.03	71.54	79.74	16.9
		FSGA	79.23	87.69	76.15	87.69	18.8
		FSACO	86.92	85.13	79.49	86.41	8.9
		FSCOA	82.05	86.41	80.25	86.41	12.77
3	JM1	Without FS	73.35	69.94	78.99	75.85	22
		FSDE	77.18	78.73	79.85	79.72	4.9
		FSPSO	75.07	72.94	79.16	79.05	8.9
		FSGA	76.36	73.29	79.62	79.83	10.3
		FSACO	79.26	79.49	79.89	79.79	3
		FSCOA	79.13	79.66	79.91	79.82	3.75
4	CM1	Without FS	75.76	80.3	77.27	83.33	38
		FSDE	86.82	89.7	83.48	88.48	18.2
		FSPSO	83.33	83.18	81.97	85	14.5
		FSGA	85.3	88.94	83.18	88.48	17.8
		FSACO	87.42	87.27	84.55	88.18	12.1
		FSCOA	88.33	83.64	84.39	88.79	15.4
5	MC1	Without FS	96.48	97.74	95.73	97.49	39
		FSDE	97.74	98.57	97.71	97.74	19.2
		FSPSO	97.56	98.49	96.31	97.49	12.4
		FSGA	97.59	98.67	97.71	97.74	19.2
		FSACO	98.02	98.34	97.74	97.76	13.4
		FSCOA	97.94	98.59	97.71	97.96	15.7
6	MC2	Without FS	76	68	92	84	40
		FSDE	87.6	90.8	95.6	96	18.4
		FSPSO	80	75.2	92.8	88.4	17.2
		FSGA	85.2	89.6	93.2	95.2	18.4
		FSACO	89.2	86	96	95.2	7.2
		FSCOA	94.4	82.4	96	96	12.9
7	PC1	Without FS	89.36	88.65	87.23	86.52	38
		FSDE	93.76	95.04	91.21	93.26	19.1
		FSPSO	90.85	92.06	89.65	89.72	16.2
		FSGA	93.48	95.04	90.64	93.48	18.9
		FSACO	93.97	93.97	92.63	92.91	10.8
		FSCOA	94.4	92.77	92.77	93.83	17.9
8	PC2	Without FS	96.64	95.3	93.96	97.32	37
		FSDE	97.79	98.66	96.98	97.58	14.7
		FSPSO	97.45	96.51	95.84	97.38	15.2
		FSGA	97.65	98.32	96.31	97.48	17.1
		FSACO	97.89	97.25	97.22	98.12	10.7
		FSCOA	97.99	97.85	97.32	98.19	12.52
9	PC3	Without FS	82.41	78.7	68.98	62.03	38
		FSDE	86.34	86.39	86.85	86.44	16.6
		FSPSO	84.77	82.92	80.83	83.38	13.5
		FSGA	85.93	86.57	86.81	86.76	17.2
		FSACO	86.71	84.44	87.08	86.82	11.6
		FSCOA	87.04	85.83	87.18	86.9	13.8
10	MW1	Without FS	78.43	74.51	76.47	80.39	38
		FSDE	87.25	87.84	83.53	88.63	13.5
		FSPSO	84.71	82.75	78.82	84.31	12.9
		FSGA	85.69	87.06	82.16	86.67	17.2
		FSACO	86.67	85.29	87.25	90.39	8.7
		FSCOA	87.45	85.68	89.02	89.8	10.7
11	PC4	Without FS	84.49	91.09	86.82	47.67	38
		FSDE	90.11	93.45	91.59	92.71	17.6
		FSPSO	86.63	92.64	89.11	86.98	14.4
		FSGA	87.6	93.53	91.74	92.49	18.6
		FSACO	91.16	92.4	91.4	91.82	14.2
		FSCOA	91.74	92.95	92.33	92.75	15.5
12	PC5	Without FS	67.06	72.59	70.55	69.39	39
		FSDE	76.33	77.73	71.57	72.57	18.4
		FSPSO	71.98	73.53	70.82	70.59	15.7
		FSGA	75.63	77.81	71.46	72.92	19.3
		FSACO	76.85	75.16	72.19	71.11	14.8
		FSCOA	78.63	77.23	72.45	72.711	16.4

The median accuracy of several classifiers forced on diverse datasets, both with and without feature selection, is shown in the table. The table also displays the average tally of the attributes selected by the respective FS approach. The classifiers were evaluated using a range of datasets and previously discussed FS techniques. The experimental findings showed that the suggested FSCOA technique exceeded the other studied FS procedures in the majority of instances. For the majority of the datasets, with the exception of KC1, KC3, JM1, and MC1, the suggested FSCOA performed best when combined with KNN. With the exception of KC1, MC1, and CM1, the bulk of the datasets showed that the recommended FSCOA worked best when paired with NB. The majority of the datasets demonstrated that the suggested FSCOA performed best when combined with QDA, with the exception of KC3, MW1, and PC5. With the exception of JM1, the majority of the datasets showed that the previously researched FS approaches outperformed the suggested FSCOA strategy when used in conjunction with DT. Similarly, applying the proposed FSCOA technique to the JM1 dataset with all the analyzed classifiers, except KNN, yielded the highest accuracy. Furthermore, the bulk of the datasets yielded the best accuracy for all examined classifiers, with the exception of DT. It is crucial to remember that the suggested FSCOA technique could only provide the best prediction using QDA and NB classifiers for the KC1 and KC3 datasets, respectively.

**Figure 3.**
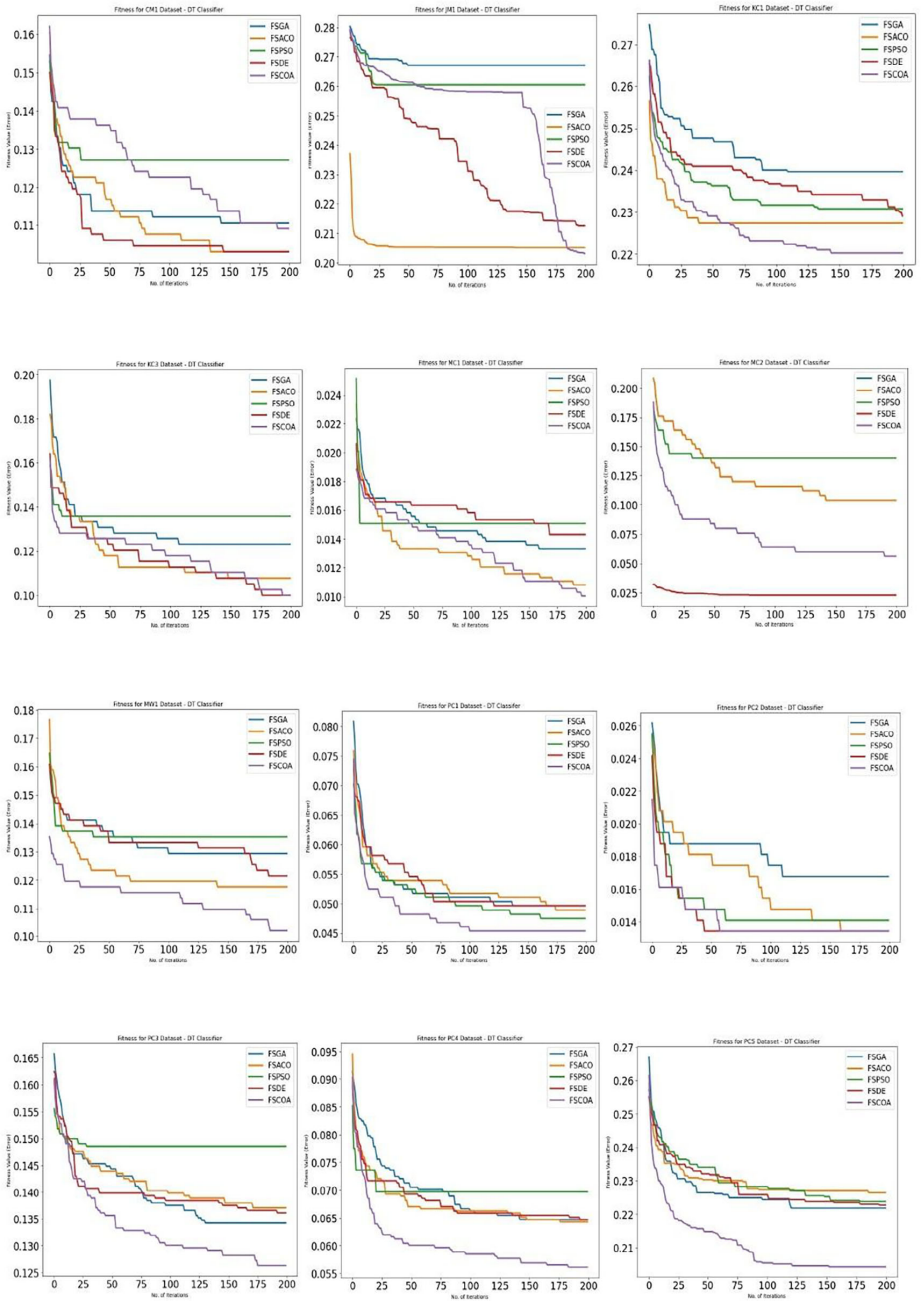
DT fitness error plot.

Figures 3 through 6 ^53^ display, respectively, the fitness error plots for the suggested FSCOA approach and other FS strategies utilizing the examined classifiers DT, KNN, NB, and QDA. Error plots of all 12 datasets are included in each graph. The error plots show that in most cases, the error plot of the suggested FSCOA is smaller than those of the other FS approaches employed in this investigation. Furthermore, the error plot of the suggested FSCOA methodology matches that of the various existing FS methods. However, the error plot of the suggested FSCOA approach exceeds that of the other FS techniques that have been evaluated under certain circumstances.

Figure 3 ^53^ shows that in most datasets, the fitness error plot of the proposed FSCOA approach using the DT classifier is smaller than that of the other FS models, with the exception of CM1, MC2, and KC3. However, for the KC3 dataset, the error plot overlapped with that of the FSDE after 190 iterations. The error plot for the CM1 dataset is located above the FSDE and FSACO. Furthermore, the plot for the MC2 dataset is above the FSDE.

**Figure 4.**
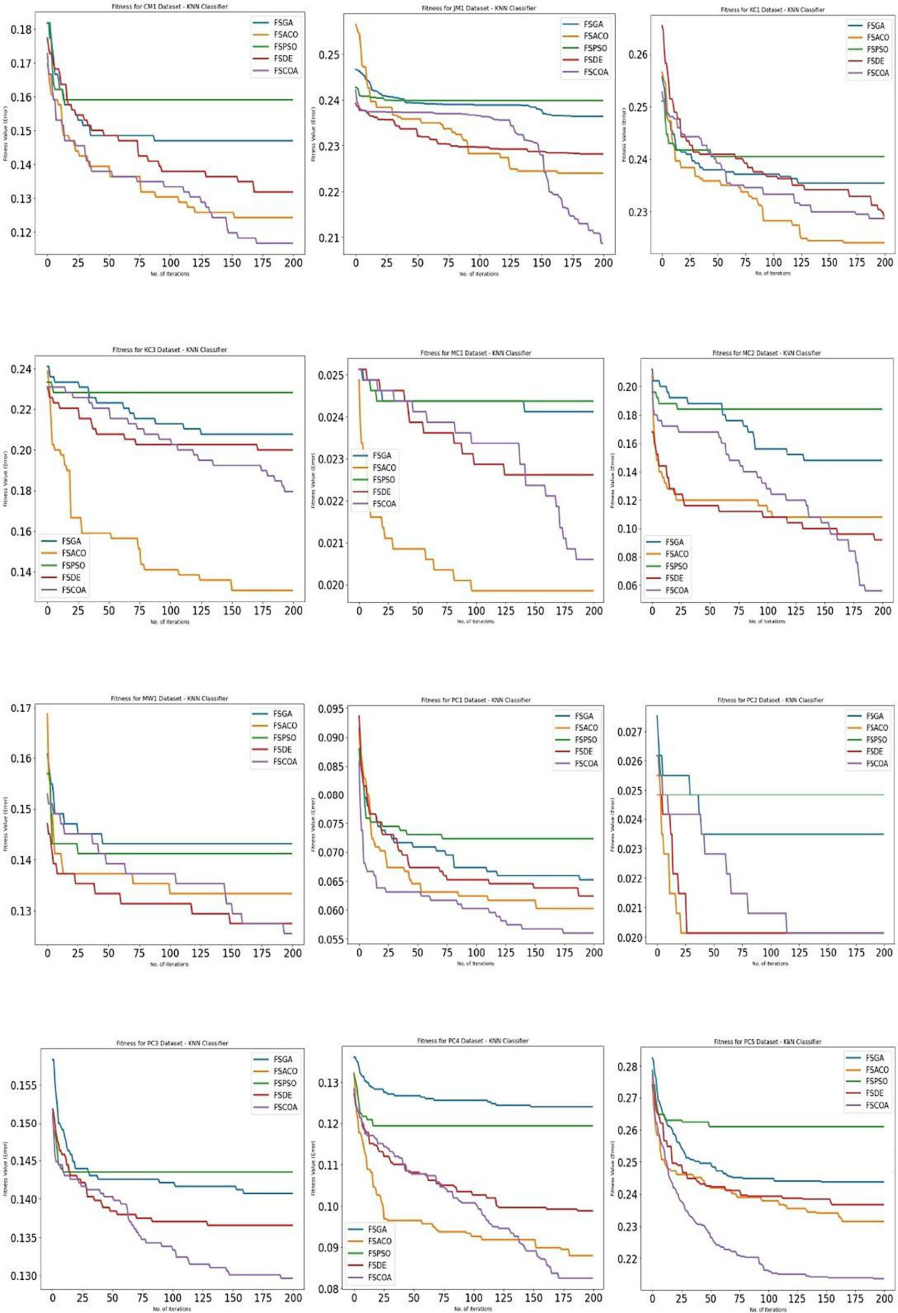
KNN fitness error plot.

The fitness error plot of the proposed FSCOA approach with the KNN classifier is lower than that of the previous FS models for most datasets, as shown in Figure 4, ^53^ with the exception of KC1, KC3, MC1, and PC2. The error plot after 115 iterations corresponds to FSDE and FSACO for the PC2 dataset, but it is above the FSACO model for the KC1, KC3, and MC1 datasets.

**Figure 5.**
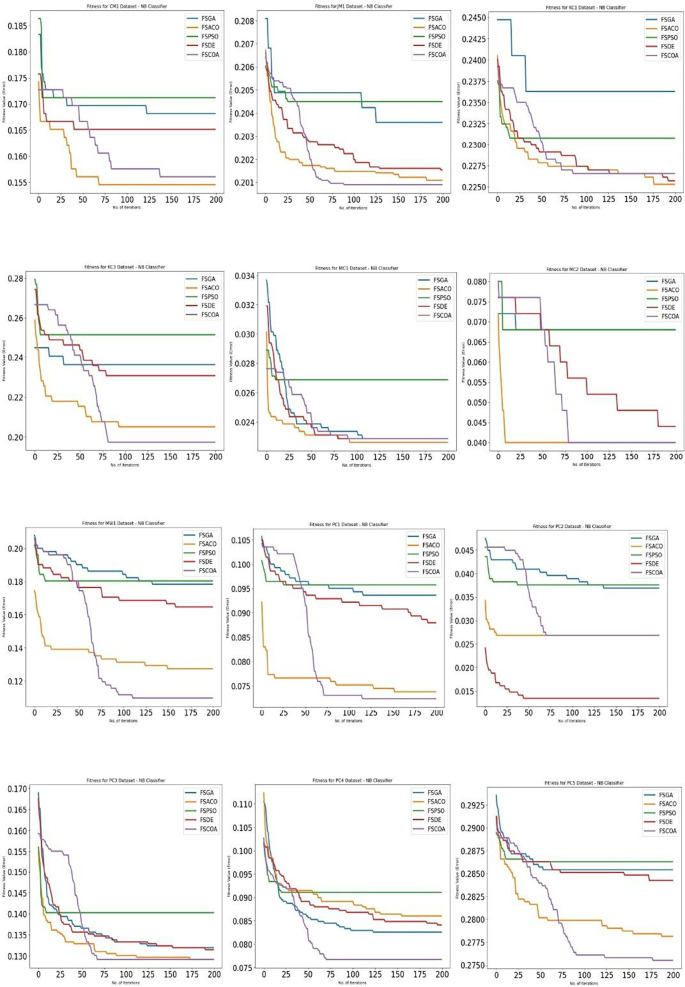
NB fitness error plot.

As shown in Figure 5, ^53^ the fitness error plot of the suggested FSCOA technique with the NB classifier was lower in the majority of datasets than that of the prior FS models, with the exception of CM1, KC1, MC1, PC2, MC2, and PC3. After 75 iterations, the error plot for the MC2 dataset matches that of FSACO. For the PC3 dataset, the error plot of the suggested FSCOA method matches that of FSACO after 175 iterations. However, for datasets CM1, KC1, and MC1, the error plot was above that of the FSACO model. Moreover, for PC2 and KC1, the plot was above the FSDE.

**Figure 6.**
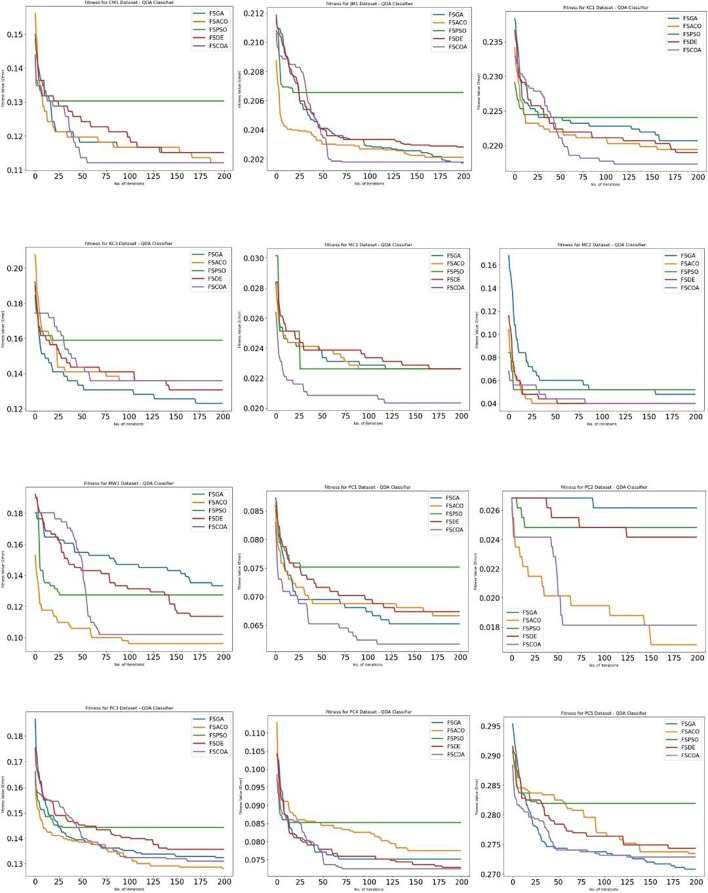
QDA fitness error plot.

Figure 6 ^53^ illustrates that for most datasets (except CM1, JM1, KC3, MW1, PC2, and PC3), the fitness error plot of the proposed FSCOA approach with the NB classifier is smaller than that of the previous FS models. Following 180 iterations, the CM1 dataset’s error plot aligns with that of the FSACO dataset. The error plot of the proposed FSCOA algorithm matches that of FSGA after 175 iterations for the JM1 dataset. The error plot is above that of the FSACO model for datasets MW1, PC2, and PC3. The graphic is also above FSGA and FSDE for the KC3 dataset. The error plot of the proposed FSCOA approach is above that of FSGA for the PC3 dataset.

The FS algorithms employed in this study use several hyperparameters. For 200 iterations, an examination was performed with a population size of 30. The crossover rate (CR) in FSGA and FSDE was maintained at 0.8 and 0.9, respectively. For FSGA, the mutation rate (MR) is 0.01. In the FSDE, the scaling factor (SF) was set to 0.8. In FSPSO, the maximum inertia weight (IWmax) and the minimum inertia weight (IWmin) have been fixed as 0.9 and 0.4 accordingly. Two were chosen as the acceleration factors. The fixed values of alpha (α) , rho (ρ) , and beta (β) in FSACO were 1, 0.2, and 0.1, respectively. The governing criterion speeds for alpha $\left({\text{Speed}}_{\alpha }\right)$ , beta $\left({\text{Speed}}_{\beta }\right)$ , and gamma $\left({\text{Speed}}_{\gamma }\right)$ were adjusted appropriately for FSCOA using the Rand function. In addition, the radiation propagation radius (rad) was similarly fixed between 0 and 1 using the rand function.

### Statistical analysis

This section provides extensive statistical scrutiny of the empirical findings of this work. Statistical analysis ^50^ is a popular method for quantifying, examining, evaluating, and drawing conclusions from data. Tests classified as parametric or non-parametric were the two breeds used for statistical analysis. A type of statistical analysis, known as parametric statistical testing, assumes that the data under study conform to a specific probability distribution, most frequently a normal distribution. Several assumptions, such as the independence of observations, homogeneity of variance, and normality, must hold true to employ parametric tests. Ensuring that the assumptions are met is essential when conducting a parametric test, because failing to do so may result in erroneous results and invalid conclusions. Therefore, it is crucial to confirm these hypotheses in advance and, if necessary, to use non-parametric validation. A type of statistical scrutiny known as non-parametric statistical testing does not depend on a specific probability distribution hypothesis for the data under study. Non-parametric validations, on the other hand, are more broadly applicable and resilient to assumption violations than parametric tests because they depend on the hierarchy or placement of the data. However, when the presumptions of the parametric validations are satisfied, they might be less effective than the latter. It is essential to choose a statistical validation suitable for an exploration topic and the properties of the data being examined. In this study, the Friedman Test, ^51^ a non-parametric rank-based test, has been carefully considered. Based on the effectiveness of the classification, each model associated with the trial was ranked according to the Friedman test. The lowest count correlates with the greatest slot and the largest count correlates with the smallest slot.

To begin with, Eq. (19) was employed to determine the average rank ( AverageRankModels ) of all graded models (FSDE, FSPSO, FSGA, FSACO, FSCOA, and Without FS), in addition to a number of classification models (KNN, DT, NB, and QDA). Table 3 ^54^ presents an illustration these findings.

$$\text{AverageRankModels}=\frac{\sum \text{RankModels}}{\text{Total number of Models}\;\left(A\right)}$$ (19)

**Table 3.** For twelve NASA datasets, the average rank of all FS algorithms (Friedman Rank).

Sl. No.	Datasets	FS Algorithms/Classifiers	KNN	DT	NB	QDA	AverageRankModels
1	KC1	Without FS	69.62 (6)	72.15 (6)	74.26 (5)	74.26 (6)	5.75
		FSDE	76.46 (4)	77.09 (1)	77.22 (3)	78.10 (2)	2.5
		FSPSO	74.64 (5)	73.12 (5)	76.12 (4)	76.27 (5)	4.75
		FSGA	76.47 (3)	76.03 (3)	77.22 (3)	77.93 (3)	3
		FSACO	77.69 (1)	76.85 (2)	77.47 (1)	77.85 (4)	2
		FSCOA	77.13 (2)	75.32 (4)	77.34 (2)	78.27 (1)	2.25
2	KC3	Without FS	74.36 (6)	76.92 (6)	66.67 (6)	76.92 (5)	5.75
		FSDE	80 (3)	90 (1)	76.92 (3)	86.92 (2)	2.25
		FSPSO	76.15 (5)	81.03 (5)	71.54 (5)	79.74 (4)	4.75
		FSGA	79.23 (4)	87.69 (2)	76.15 (4)	87.69 (1)	2.75
		FSACO	86.92 (1)	85.13 (4)	79.49 (2)	86.41 (3)	2.5
		FSCOA	82.05 (2)	86.41 (3)	80.25 (1)	86.41 (3)	2.25
3	JM1	Without FS	73.35 (6)	69.94 (6)	78.99 (6)	75.85 (6)	6
		FSDE	77.18 (3)	78.73 (3)	79.85 (3)	79.72 (4)	3.25
		FSPSO	75.07 (5)	72.94 (5)	79.16 (5)	79.05 (5)	5
		FSGA	76.36 (4)	73.29 (4)	79.62 (4)	79.83 (1)	3.25
		FSACO	79.26 (1)	79.49 (2)	79.89 (2)	79.79 (3)	2
		FSCOA	79.13 (2)	79.66 (1)	79.91 (1)	79.82 (2)	1.5
4	CM1	Without FS	75.76 (6)	80.30 (6)	77.27 (6)	83.33 (5)	5.75
		FSDE	86.82 (3)	89.70 (1)	83.48 (3)	88.48 (2)	2.25
		FSPSO	83.33 (5)	83.18 (5)	81.97 (5)	85 (4)	4.75
		FSGA	85.30 (4)	88.94 (2)	83.18 (4)	88.48 (2)	3
		FSACO	87.42 (2)	87.27 (3)	84.55 (1)	88.18 (3)	2.25
		FSCOA	88.33 (1)	83.64 (4)	84.39 (2)	88.79 (1)	2
5	MC1	Without FS	96.48 (6)	97.74 (6)	95.73 (4)	97.49 (5)	5.25
		FSDE	97.74 (3)	98.57 (3)	97.71 (2)	97.74 (3)	2.75
		FSPSO	97.56 (5)	98.49 (4)	96.31 (3)	97.49 (4)	4
		FSGA	97.59 (4)	98.67 (1)	97.71 (2)	97.74 (3)	2.5
		FSACO	98.02 (1)	98.34 (5)	97.74 (1)	97.76 (2)	2.25
		FSCOA	97.94 (2)	98.59 (2)	97.71 (2)	97.96 (1)	1.75
6	MC2	Without FS	76 (6)	68 (6)	92 (5)	84 (4)	5.25
		FSDE	87.6 (3)	90.8 (1)	95.6 (2)	96 (1)	1.75
		FSPSO	80 (5)	75.2 (5)	92.8 (4)	88.4 (3)	4.25
		FSGA	85.2 (4)	89.6 (2)	93.2 (3)	95.2 (2)	2.75
		FSACO	89.2 (2)	86 (3)	96 (1)	95.2 (2)	2
		FSCOA	94.4 (1)	82.4 (4)	96 (1)	96 (1)	1.75
7	PC1	Without FS	89.36 (6)	88.65 (5)	87.23 (6)	86.52 (6)	5.75
		FSDE	93.76 (3)	95.04 (1)	91.21 (3)	93.26 (3)	2.5
		FSPSO	90.85 (5)	92.06 (4)	89.65 (5)	89.72 (5)	4.75
		FSGA	93.48 (4)	95.04 (1)	90.64 (4)	93.48 (2)	2.75
		FSACO	93.97 (2)	93.97 (2)	92.63 (2)	92.91 (4)	2.5
		FSCOA	94.40 (1)	92.77 (3)	92.77 (1)	93.83 (1)	1.5
8	PC2	Without FS	96.64 (6)	95.30 (6)	93.96 (6)	97.32 (6)	6
		FSDE	97.79 (3)	98.66 (1)	96.98 (3)	97.58 (3)	2.5
		FSPSO	97.45 (5)	96.51 (5)	95.84 (5)	97.38 (5)	5
		FSGA	97.65 (4)	98.32 (2)	96.31 (4)	97.48 (4)	3.5
		FSACO	97.89 (2)	97.25 (4)	97.22 (2)	98.12 (2)	2.5
		FSCOA	97.99 (1)	97.85 (3)	97.32 (1)	98.19 (1)	1.5
9	PC3	Without FS	82.41 (6)	78.70 (6)	68.98 (6)	62.03 (6)	6
		FSDE	86.34 (3)	86.39 (2)	86.85 (3)	86.44 (4)	3
		FSPSO	84.77 (5)	82.92 (5)	80.83 (5)	83.38 (5)	5
		FSGA	85.93 (4)	86.57 (1)	86.81 (4)	86.76 (3)	3
		FSACO	86.71 (2)	84.44 (4)	87.08 (2)	86.82 (2)	2.5
		FSCOA	87.04 (1)	85.83 (3)	87.18 (1)	86.90 (1)	1.5
10	MW1	Without FS	78.43 (6)	74.51 (6)	76.47 (6)	80.39 (6)	6
		FSDE	87.25 (2)	87.84 (1)	83.53 (3)	88.63 (3)	2.25
		FSPSO	84.71 (5)	82.75 (5)	78.82 (5)	84.31 (5)	5
		FSGA	85.69 (4)	87.06 (2)	82.16 (4)	86.67 (4)	3.5
		FSACO	86.67 (3)	85.29 (4)	87.25 (2)	90.39 (1)	2.5
		FSCOA	87.45 (1)	85.68 (3)	89.02 (1)	89.80 (2)	1.75
11	PC4	Without FS	84.49 (6)	91.09 (6)	86.82 (6)	47.67 (6)	6
		FSDE	90.11 (3)	93.45 (2)	91.59 (3)	92.71 (2)	2.5
		FSPSO	86.63 (5)	92.64 (4)	89.11 (5)	86.98 (5)	4.75
		FSGA	87.60 (4)	93.53 (1)	91.74 (2)	92.49 (3)	2.5
		FSACO	91.16 (2)	92.40 (5)	91.40 (4)	91.82 (4)	3.75
		FSCOA	91.74 (1)	92.95 (3)	92.33 (1)	92.75 (1)	1.5
12	PC5	Without FS	67.06 (6)	72.59 (6)	70.55 (6)	69.39 (6)	6
		FSDE	76.33 (3)	77.73 (2)	71.57 (3)	72.57 (3)	2.75
		FSPSO	71.98 (5)	73.53 (5)	70.82 (5)	70.59 (5)	5
		FSGA	75.63 (4)	77.81 (1)	71.46 (4)	72.92 (1)	2.5
		FSACO	76.85 (2)	75.16 (4)	72.19 (2)	71.11 (4)	3
		FSCOA	78.63 (1)	77.23 (3)	72.45 (1)	72.71 (2)	1.75

Table 4 ^54^ summarizes the findings of grading the median of all ranks of diversified amalgamation setups (FSDE, FSPSO, FSGA, FSACO, FSCOA, and Without FS) for all the datasets used in Eq. (20).

$$\text{AverageRankDatasets}=\frac{\sum \text{AverageRankModels}}{\text{Total number of Datasets}\;\left(B\right)}$$ (20)

**Table 4.** AvgRank of all FS configurations.

Sl. No.	Datasets	Without FS	FSDE	FSPSO	FSGA	FSACO	FSCOA
1	KC1	5.75	2.5	4.75	3	2	2.25
2	KC3	5.75	2.25	4.75	2.75	2.5	2.25
3	JM1	6	3.25	5	3.25	2	1.5
4	CM1	5.75	2.25	4.75	3	2.25	2
5	MC1	5.25	2.75	4	2.5	2.25	1.75
6	MC2	5.25	1.75	4.25	2.75	2	1.75
7	PC1	5.75	2.5	4.75	2.75	2.5	1.5
8	PC2	6	2.5	5	3.5	2.5	1.5
9	PC3	6	3	5	3	2.5	1.5
10	MW1	6	2.25	5	3.5	2.5	1.75
11	PC4	6	2.5	4.75	2.5	3.75	1.5
12	PC5	6	2.75	5	2.5	3	1.75
	Average	5.8	2.52	4.75	2.92	2.48	1.75
	RankDatasets	AvgRank6	AvgRank3	AvgRank5	AvgRank4	AvgRank2	AvgRank1

The following are the average ranks for all the correlated configurations included in this observation. {AvgRank1=1.75,AvgRank2=2.48,AvgRank3=2.52,AvgRank4=2.92,AvgRank5=4.75,AvgRank6=5.8} . The median ranks of the models were employed to gauge the $X_{F}$ statistics, referred to as $X_{F}^{2}$ using Eq. (21) and a presented value of 23.29.

$$X_{F}^{2}=\frac{12\times B}{A\times \left(A+1\right)}\times \left[\sum_{i=1}^{6}{\left(\text{AvgRank}\left(i\right)\right)}^{2}-\frac{A\times {\left(A+1\right)}^{2}}{4}\;\right]\;$$ (21)

Twelve datasets ( *B*=12) and six models ( *A*=6) were considered in this experiment. The Friedman statistic ( $F_{F}$ ) value was computed using Eq. (22) using ( *B* − 1) and $X_{F}^{2}$ .

$$F_{F}=\frac{\left(B-1\right)\times X_{F}^{2}}{B\times \left(A-1\right)-X_{F}^{2}}$$ (22)

The value of ( $F_{F}$ ) estimated to be 6.978. The critical value was determined as 2.383 by employing the degrees of freedom as (6 − 1 = 5) × (12 − 1 = 11) and (6 − 1 = 5), with α = 0.05, as the significance level. Given that the critical value of 2.383 is smaller than that of the Friedman statistic ( $F_{F}$ = 6.978), the null hypothesis is rejected. It also determines whether to adopt an alternate theory. This implies that two or more configurations are distinct from one another. The Holm method is usually employed to investigate the Post Hoc test after the null hypothesis is jilted and the substitute hypothesis is endorsed. By employing the Holm technique, the pvalue and z−value were applied to assess how well each distinct model performed relative to the other models. ^52^ Eq. (23) was used to obtain the value of *z*. The z−value and normal distribution table were used to calculate the value of p .

$$z=\frac{\text{AvgRank}\left(i\right)-\text{AvgRank}\left(j\right)}{\sqrt{\frac{A\times \left(A+1\right)}{6\times B}}}$$ (23)

In this experiment, the terminologies B, A, and z represent the number of datasets, number of configurations employed in this investigation, and value of *z*, respectively. The terms AvgRank(i) and AvgRank(j) represents the average rank of $i^{\mathrm{th}}$ and $j^{\mathrm{th}}$ model, respectively. The pvalue , z−valu e, and α/(A−i) of the recommended configurations were compared, and Table 5 summarizes the findings. For this particular instance, we set the significance level, α, at 0.05.

Table 5 ^54^ illustrates that in most cases, the p-value is lower than or equivalent to the value of α/(A−i) with the exception of the FSCOA and FSGA models and FSCOA and FSACO models. It resolves that the FSCOA model is statistically noteworthy and attains a superior dossier when matched to other configurations, excluding the FSGA and FSACO models. However, there was no statistically significant variation in the performances of these models.

**Table 5.** Holm procedure.

Sl. No.	Model used in FS	z-value	p-value	α/(A−i)
1	FSCOA: without FS	5.307	0.00001	0.01
2	FSCOA: FSGA	1.533	0.06	0.0125
3	FSCOA: FSDE	1.009	0.156	0.0166
4	FSCOA: FSPSO	3.931	0.000042	0.025
5	FSCOA: FSACO	0.956	0.16	0.05

## Conclusion

This study proposed a novel FS approach called FSCOA, which applies a meta-heuristic procedure called CDO. The proposed FSCOA technique exhibits nuclear core reactor disruption to determine the best set of attributes by carefully discarding irrelevant or insignificant ones. This study investigates the impact of the proposed FSCOA approach on 12 publicly available NASA datasets by employing four widely used classifiers (DT, KNN, NB, and QDA). One elementary purpose was to correlate the predictive behavior of the proposed FSCOA approach with other existing FS techniques, namely, FSDE, FSPSO, FSACO, and FSGA. The Friedman test was used to test the statistical validity of the proposed FSCOA method. The test outcome showed that at least two models were significantly different, leading to the repudiation of the null hypothesis. This necessitates the use of the Holm test. The experimental findings suggested that the proposed FSCOA approach demonstrated higher performance when selecting the optimal set of features correlated to the studied FS procedures. However, the behavior of the proposed FSCOA approach may vary across different datasets and classifiers. In the future, we aim to expand the scope of this research by employing real-world project datasets. We also look forward to investigating the efficiency of the suggested FSCOA approach by increasing the number and variety of classifiers, especially ensemble classifiers, and employing more optimization algorithms for feature selection.

## Data Availability

Figshare: Anand, Kunal (2024). Dataset 1: Zip file containing the underlying data of the presented methods and results in jpeg files. figshare. Figure. https://doi.org/10.6084/m9.figshare.25681782.v1. ^53^ This dataset contains the following underlying data:
•
Figure 1. Blueprint of the suggested FSCOA methodology.jpg •
Figure 2. Flow-diagram of recommended FSCOA approach.jpg •
Figure 3. DT fitness error plot.jpg •
Figure 4. KNN fitness error plot.jpg •
Figure 5. NB fitness error plot.jpg •
Figure 6. QDA fitness error plot.jpg Figshare: Anand, Kunal (2024). Dataset 2 Zip file containing underlying data of the presented results in csv files. figshare. Dataset. https://doi.org/10.6084/m9.figshare.25683600.v1 ^54^ This dataset contains the following underlying data:
•
Table 2 Accuracy percentage and number of features selected.csv •
Table 3 Average rank of all FS algorithms for twelve NASA datasets.csv •
Table 4. AvgRank of all FS configurations.csv\ •
Table 5. Holm procedure.csv The data are available under the terms of the Creative Commons Attribution 4.0 International license (CC-BY 4.0).
