# Supplementary material for: Implementation of Chernobyl disaster optimizer based feature selection approach to predict software defects
Source: F1000Res. 2024 Dec 17;13:844. Originally published 2024 Jul 29. [Version 2] doi: 10.12688/f1000research.150927.2 (PMC11809630; doi:10.12688/f1000research.150927.2)
Supplement: Peer review history

# Reviewer report: Reviewer response for version 2

**DOI:** 10.5256/f1000research.175264.r349537

**Published:** 2024-12-26

**Authors:** Francis Palma

**Affiliations:** 1 University of New Brunswick Fredericton, Fredericton, New Brunswick, Canada

I can propose to index the manuscript in the current form.

Is the work clearly and accurately presented and does it cite the current literature?

Yes

If applicable, is the statistical analysis and its interpretation appropriate?

Yes

Are all the source data underlying the results available to ensure full reproducibility?

Yes

Is the study design appropriate and is the work technically sound?

Yes

Are the conclusions drawn adequately supported by the results?

Yes

Are sufficient details of methods and analysis provided to allow replication by others?

Partly

Reviewer Expertise:

NA

I confirm that I have read this submission and believe that I have an appropriate level of expertise to confirm that it is of an acceptable scientific standard.

# Response

**Published:** 2024-12-28

**Authors:** Kunal Anand

**Affiliations:** School of Computer Engineering, Kalinga Institute of Industrial Technology, Bhubaneswar, Odisha, India

Respected Reviewer,

 I want to express my gratitude for looking over my research paper. I sincerely appreciate the time and effort you took to review my work and offer helpful criticism that improved the final manuscript's quality. I am really encouraged by your acceptance of the work, and your helpful criticism helped me to improve the impact and clarity of my research. Once more, I want to thank you for your invaluable contributions to my work. Regards

# Reviewer report: Reviewer response for version 2

**DOI:** 10.5256/f1000research.175264.r349536

**Published:** 2024-12-18

**Authors:** Ahmed Abdu

**Affiliations:** 1 Northwestern Polytechnical University, Xi’an, China

All the concerns and suggestions I outlined in my previous review have been satisfactorily addressed.

Is the work clearly and accurately presented and does it cite the current literature?

Partly

If applicable, is the statistical analysis and its interpretation appropriate?

No

Are all the source data underlying the results available to ensure full reproducibility?

No source data required

Is the study design appropriate and is the work technically sound?

Partly

Are the conclusions drawn adequately supported by the results?

Yes

Are sufficient details of methods and analysis provided to allow replication by others?

No

Reviewer Expertise:

Software engineering, software defect prediction, deep learning

I confirm that I have read this submission and believe that I have an appropriate level of expertise to confirm that it is of an acceptable scientific standard.

# Response

**Published:** 2024-12-28

**Authors:** Kunal Anand

**Affiliations:** School of Computer Engineering, Kalinga Institute of Industrial Technology, Bhubaneswar, Odisha, India

Esteemed Reviewer,

 I would like to sincerely thank you for reviewing my research paper. I deeply appreciate the time and effort you invested in evaluating my work and providing insightful comments that enhanced the quality of the final manuscript. Your acceptance of the paper is a tremendous encouragement to me, and your constructive feedback was instrumental in refining the clarity and impact of my research. Thank you once again for your valuable contributions to my work. Regards

# Reviewer report: Reviewer response for version 1

**DOI:** 10.5256/f1000research.165541.r319618

**Published:** 2024-09-20

**Authors:** Francis Palma

**Affiliations:** 1 University of New Brunswick Fredericton, Fredericton, New Brunswick, Canada

The article is sound and complete regarding technical contributions. However, some clarifications and methodological discussions are missing. Below are my more detailed comments.

 1. In the Abstract, the authors said, "To overcome the above shortcomings, this study aims to develop an innovative FS

 technique, namely, the Chernobyl Optimization Algorithm (FSCOA), to unwrap the most informative features" -> I assume the authors are not developing COA? Rather applying or using it? This phrase needs to be revised.

 2. The first line in the Methods part of the Abstract: "attacking" -> "attaching".

 3. The last paragraph of the Introduction provides the outline of the article. However, later, I found no section numbers or identifiers. Shouldn't the sections be numbered?

 4. The Related Works section discusses 11 studies. I would expect a table providing a summary and comparison among these studies, including your own study (this one).

 5. The overall methodology should be presented as various Steps in a more systematic, organized, and modular manner...

 6. The three steps mentioned in Page 6, I suggest you show these steps in the figure using like Step 1, Step 2, etc.

 7. In Figure 1, not sure why this is called a FSCOA approach, because feature selection is part of any model building activity, you are just using a new technique for feature selection... right?

 8. In Figure 1, the testing data is not fed to the model training phase; it is generally fed to the model testing phase, right? The figure needs a revision.

 9. The figures e.g. Fig 1 and 2 in general look of poor quality, this is perhaps the authors did not use vector/scalable images, and simple converted images to pdf from MS Word affects the figure quality.

 10. "Alpha fragment" title on page 9 should be in bold.

 11. Algorithm 1 on page 11, how is this related to Figure 2? Have you also described the Algorithm 1 in this article? If not, the authors should briefly discuss the various Algorithmic steps, at least at the high level.

 12. In Figures 3 to 6, the x and y-axis labels are not legible. Should the font size be increased?

 13. Before the Conclusion section, this article is missing one major section: "Threats to Validity." What are the threats to the validity of your results? The authors should discuss various internal, external, construct, reliability, etc. threats.

Is the work clearly and accurately presented and does it cite the current literature?

Yes

If applicable, is the statistical analysis and its interpretation appropriate?

Yes

Are all the source data underlying the results available to ensure full reproducibility?

Yes

Is the study design appropriate and is the work technically sound?

Yes

Are the conclusions drawn adequately supported by the results?

Yes

Are sufficient details of methods and analysis provided to allow replication by others?

Partly

Reviewer Expertise:

Software engineering, software quality, software maintenance and evolution.

I confirm that I have read this submission and believe that I have an appropriate level of expertise to confirm that it is of an acceptable scientific standard, however I have significant reservations, as outlined above.

# Response

**Published:** 2024-11-25

**Authors:** Kunal Anand

**Affiliations:** School of Computer Engineering, Kalinga Institute of Industrial Technology, Bhubaneswar, Odisha, India

1. **In the Abstract, the authors said, "To overcome the above shortcomings, this study aims to develop an innovative FS technique, namely, the Chernobyl Optimization Algorithm (FSCOA), to unwrap the most informative features" -> I assume the authors are not developing COA? Rather applying or using it? This phrase needs to be revised.**

**Response:** We sincerely thank the distinguished reviewer for their insightful feedback on our work. In this regard, we would like to inform that the above mentioned phrase has been reworked in the revised manuscript.

  

1. **The first line in the Methods part of the Abstract: "attacking" -> "attaching".**

**Response:** We truly value the insightful reviewer's feedback on our work. Regarding this, we would like to say that the complete sentence has been reworked and also has been removed from the method part to the background part in the abstract. Here, the word “attacking” has been replaced with “attaching” in the revised manuscript.

  

1. **The last paragraph of the Introduction provides the outline of the article. However, later, I found no section numbers or identifiers. Shouldn't the sections be numbered?**

**Response:** We are deeply appreciative of the esteemed reviewer's perceptive comments on our work. Regarding this, we want to submit that the section numbers for the paper's headers were included in the original manuscript when it was submitted. Nevertheless, the document was published using the publisher's template, which omitted the section numbers. The final paragraph has been rewritten in the updated text to adhere to the distinguished reviewer's recommendation.

  

1. **The Related Works section discusses 11 studies. I would expect a table providing a summary and comparison among these studies, including your own study (this one).**

**Response:** We sincerely appreciate the insightful feedback on our efforts provided by the excellent reviewer. In light of this, we would like to submit that, as desired by the distinguished reviewer, the literature review is included as a table at the conclusion of the related work section. We further want to state that the proposed work has been appropriately compared with the existing FS techniques used in the referenced studies in the result analysis section.

  

1. **The overall methodology should be presented as various Steps in a more systematic, organized, and modular manner…**

**Response:** We truly value the thoughtful criticism of our work that the outstanding reviewer gave us. Considering this, we would like to submit that the proposed methodology section has been reworked in line with the esteemed reviewer’s advise in the revised manuscript.

  

1. **The three steps mentioned in Page 6, I suggest you show these steps in the figure using like Step 1, Step 2, etc.**

**Response:** The outstanding reviewer's informative comments on our efforts are greatly appreciated. Accordingly, we would like to state that the introductory paragraph in the section “Feature selection using based on Chernobyl Disaster Optimization Algorithm” has been revised.

  

1. **In Figure 1, not sure why this is called a FSCOA approach, because feature selection is part of any model building activity, you are just using a new technique for feature selection... right?**

**Response:** We sincerely appreciate the insightful critique of our work provided by the esteemed reviewer. In light of this, we would like to state that the figure 1 has been revised in line with the esteemed reviewer's suggestion.

  

1. **In Figure 1, the testing data is not fed to the model training phase; it is generally fed to the model testing phase, right? The figure needs a revision.**

**Response:** The distinguished reviewer's perceptive criticism of our work is greatly appreciated. Given this, we would like to state that the figure 1 has been updated to reflect the reviewer's insightful recommendations.

  

1. **The figures e.g. Fig 1 and 2 in general look of poor quality, this is perhaps the authors did not use vector/scalable images, and simple converted images to pdf from MS Word affects the figure quality.**

**Response:** We are grateful for the insightful critique of our work provided by the esteemed reviewer. In light of this, we would like to state that the reviewer's wise suggestions have been incorporated into the improved quality of Figures 1 and 2.

  

1. **"Alpha fragment" title on page 9 should be in bold.**

**Response:** The distinguished reviewer's perceptive criticism of our work is greatly appreciated. Taking this into consideration, we are pleased to submit that the reviewer's insightful recommendations have been implemented.

  

1. **Algorithm 1 on page 11, how is this related to Figure 2? Have you also described the Algorithm 1 in this article? If not, the authors should briefly discuss the various Algorithmic steps, at least at the high level.**

**Response:** The distinguished reviewer's perceptive criticism of our work is greatly appreciated. In light of this, we would like to submit that algorithm 1 is shown graphically in Figure 2. The text and the alpha, beta, and gamma segments that follow figure 2 provide a succinct explanation of algorithm 1 and figure 2.

  

1. **In Figures 3 to 6, the x and y-axis labels are not legible. Should the font size be increased?**

**Response:** We are grateful for the insightful critique of our work provided by the esteemed reviewer. Given this, we would like to state that the font sizes of the individual images in figures 3 through 6 are appropriate. However, while retaining individual photographs, the paper's length was increasing. For this reason, the only reason to combine the images on a smaller scale was to reduce the paper's length.

  

1. **Before the Conclusion section, this article is missing one major section: "Threats to Validity." What are the threats to the validity of your results? The authors should discuss various internal, external, construct, reliability, etc. threats.**

**Response:** We sincerely appreciate the insightful critique of our work provided by the exceptional reviewer. In light of this, we would like to state that the updated paper now includes a new “Threats to Validity” section that complies with the esteemed reviewer's wise recommendation.

# Reviewer report: Reviewer response for version 1

**DOI:** 10.5256/f1000research.165541.r319612

**Published:** 2024-09-17

**Authors:** Ahmed Abdu

**Affiliations:** 1 Northwestern Polytechnical University, Xi’an, China

This paper presents an approach to addressing the challenges of software defect prediction (SDP) through the innovative use of the Chornobyl Optimization Algorithm (COA) for feature selection. The problem of handling high-dimensional datasets in SDP is well-recognized, and the authors' focus on reducing these dimensions through feature selection techniques is highly relevant to improving predictive performance. The abstract provides a clear overview of the limitations of existing meta-heuristic algorithms, positioning the COA as a promising alternative. However, it requires some major revisions to fully meet the expectations for clarity and depth in the current research context. Addressing these revisions will help strengthen the study's overall contribution and impact:

 1. The abstract should clarify the specific contribution of the Chernobyl Optimization Algorithm (FSCOA) in comparison to existing methods. The abstract mentions drawbacks of other meta-heuristic algorithms but doesn't specify how FSCOA addresses these issues in a distinct or innovative way. Adding a brief statement on the unique advantages of FSCOA (e.g., better performance, reduced computational cost, or improved convergence) would enhance clarity and highlight the novelty of the study.

 2. The introduction section outlines the research focus, but the main contributions of the paper should be revised to clearly delineate three distinct and understandable contributions. Clarifying these contributions will help readers better grasp the novel aspects of your work and its significance in advancing the field.

 3. The authors should consider extending the Related Work section by including more recent references to ensure the study is up-to-date with the latest advancements in software defect prediction. Specifically, citing the following studies would strengthen the discussion of deep learning and feature selection approaches in software defect prediction:

- Abdu, A., Zhai, Z., Abdo, H. A., & Algabri, R. (2024). Software Defect Prediction Based on Deep Representation Learning of Source Code From Contextual Syntax and Semantic Graph. IEEE Transactions on Reliability.
- Abdu, A., Zhai, Z., Abdo, H. A., Algabri, R., & Lee, S. (2023). Graph-Based Feature Learning for Cross-Project Software Defect Prediction. Computers, Materials and Continua, 77(1).

- Abdu, A., Zhai, Z., Abdo, H. A., Algabri, R., Al-Masni, M. A., Muhammad, M. S., & Gu, Y. H. (2024). Semantic and traditional feature fusion for software defect prediction using hybrid deep learning model. Scientific Reports, 14(1), 14771.
- Abdu, A., Zhai, Z., Algabri, R., Abdo, H. A., Hamad, K., & Al-antari, M. A. (2022). Deep learning-based software defect prediction via semantic key features of source code—systematic survey. Mathematics, 10(17), 3120.

By incorporating these studies, the authors will enrich the context of their research and ensure that their work is grounded in the most current literature on the topic.

 4. The results discussion is currently lacking depth and does not sufficiently highlight the strengths of the proposed method. The comparison with other approaches is very limited, which weakens the impact of your findings. I recommend expanding the explanation and discussion of the results, focusing on:

- How your proposed method outperforms or differs from the baseline methods.
- Highlighting key insights and practical implications of your findings.

By thoroughly discussing these aspects, you will better demonstrate the novelty and effectiveness of your method, making the results more compelling for the readers.

Is the work clearly and accurately presented and does it cite the current literature?

Partly

If applicable, is the statistical analysis and its interpretation appropriate?

No

Are all the source data underlying the results available to ensure full reproducibility?

No source data required

Is the study design appropriate and is the work technically sound?

Partly

Are the conclusions drawn adequately supported by the results?

Yes

Are sufficient details of methods and analysis provided to allow replication by others?

No

Reviewer Expertise:

Software engineering, software defect prediction, deep learning

I confirm that I have read this submission and believe that I have an appropriate level of expertise to confirm that it is of an acceptable scientific standard, however I have significant reservations, as outlined above.

## References

[1] : Semantic and traditional feature fusion for software defect prediction using hybrid deep learning model. *Sci Rep* .2024;14(1) : 10.1038/s41598-024-65639-4 14771 10.1038/s41598-024-65639-4 38951608 PMC11217391

[2] : Software Defect Prediction Based on Deep Representation Learning of Source Code From Contextual Syntax and Semantic Graph.

[3] : Deep Learning-Based Software Defect Prediction via Semantic Key Features of Source Code—Systematic Survey.

[4] : Graph-Based Feature Learning for Cross-Project Software Defect Prediction.

# Response

**Published:** 2024-11-25

**Authors:** Kunal Anand

**Affiliations:** School of Computer Engineering, Kalinga Institute of Industrial Technology, Bhubaneswar, Odisha, India

1. **The abstract should clarify the specific contribution of the Chernobyl Optimization Algorithm (FSCOA) in comparison to existing methods. The abstract mentions drawbacks of other meta-heuristic algorithms but doesn't specify how FSCOA addresses these issues in a distinct or innovative way. Adding a brief statement on the unique advantages of FSCOA (e.g., better performance, reduced computational cost, or improved convergence) would enhance clarity and highlight the novelty of the study.**

**Response:** We really appreciate the insightful reviewer's feedback on our work, and in light of this, we would like to propose that the abstract has been amended in the revised article in accordance with the reviewer's suggestions.

  

1. **The introduction section outlines the research focus, but the main contributions of the paper should be revised to clearly delineate three distinct and understandable contributions. Clarifying these contributions will help readers better grasp the novel aspects of your work and its significance in advancing the field.**

**Response:** Our sincere gratitude goes out to the renowned reviewer for his insightful feedback on our work. This is an extremely astute suggestion from the esteemed critic. As suggested by the esteemed reviewer, the major contributions of the paper have been reworked in the revised manuscript.

**3. The authors should consider extending the Related Work section by including more recent references to ensure the study is up-to-date with the latest advancements in software defect prediction. Specifically, citing the following studies would strengthen the discussion of deep learning and feature selection approaches in software defect prediction:**

**Abdu, A., Zhai, Z., Abdo, H. A., & Algabri, R. (2024). Software Defect Prediction Based on Deep Representation Learning of Source Code From Contextual Syntax and Semantic Graph. IEEE Transactions on Reliability.**

**Abdu, A., Zhai, Z., Abdo, H. A., Algabri, R., & Lee, S. (2023). Graph-Based Feature Learning for Cross-Project Software Defect Prediction. Computers, Materials and Continua, 77(1).**

**Abdu, A., Zhai, Z., Abdo, H. A., Algabri, R., Al-Masni, M. A., Muhammad, M. S., & Gu, Y. H. (2024). Semantic and traditional feature fusion for software defect prediction using hybrid deep learning model. Scientific Reports, 14(1), 14771.**

**Abdu, A., Zhai, Z., Algabri, R., Abdo, H. A., Hamad, K., & Al-antari, M. A. (2022). Deep learning-based software defect prediction via semantic key features of source code—systematic survey. Mathematics, 10(17), 3120.**

**By incorporating these studies, the authors will enrich the context of their research and ensure that their work is grounded in the most current literature on the topic.**

**Response:** We sincerely thank the distinguished critic for his insightful feedback on our work. The renowned reviewer has made a very wise recommendation. In accordance with the most recent developments in software defect prediction, a few recent works have been added to the related work area as suggested by the esteemed reviewer.

**4. The results discussion is currently lacking depth and does not sufficiently highlight the strengths of the proposed method. The comparison with other approaches is very limited, which weakens the impact of your findings. I recommend expanding the explanation and discussion of the results, focusing on:**

**How your proposed method outperforms or differs from the baseline methods.**

**Highlighting key insights and practical implications of your findings.**

**By thoroughly discussing these aspects, you will better demonstrate the novelty and effectiveness of your method, making the results more compelling for the readers.**

**Response:** We sincerely thank the distinguished reviewer for his insightful critique of our work. The recommendation from the renowned reviewer is really astute. As suggested by the respected reviewer, we have incorporated in the updated text the notable distinctions between the suggested method and the other baseline FS approaches employed in this study. Additionally, the consequences of the suggested procedures are incorporated into the updated manuscript as a distinct section called "Threats to validity."   

# Reviewer report: Reviewer response for version 1

**DOI:** 10.5256/f1000research.165541.r309141

**Published:** 2024-08-14

**Authors:** Shabib Aftab

**Affiliations:** 1 Virtual University of Pakistan, Lahore, Punjab, Pakistan

**Abstract:** The abstract should offer a succinct overview of the research paper, covering the research problem, objectives, methodology, key findings, and conclusions. It must be clear and informative, providing readers with an understanding of the study's importance. You should precisely highlight the main contributions, including the datasets utilized and the accuracy achieved by your proposed system.

**Introduction:** The introduction section should conclude with a brief outline of the structure for the remainder of the paper

 .

**Literature Review:** A distinct section dedicated to the Literature Review should be included in the paper in the form of a table.

**Results:** The results section should clearly and systematically present the study's findings. It should provide detailed information, including a thorough comparison with other related techniques. You also have to explore other measures as well in the comparison other techniques.

**Conclusion:** This section should offer a comprehensive summary of the research and include a detailed discussion of future research directions.

**English:** The paper should be reviewed to ensure the English language is free of grammatical errors.

Is the work clearly and accurately presented and does it cite the current literature?

Partly

If applicable, is the statistical analysis and its interpretation appropriate?

Partly

Are all the source data underlying the results available to ensure full reproducibility?

Yes

Is the study design appropriate and is the work technically sound?

No

Are the conclusions drawn adequately supported by the results?

Yes

Are sufficient details of methods and analysis provided to allow replication by others?

Partly

Reviewer Expertise:

software defect prediction

I confirm that I have read this submission and believe that I have an appropriate level of expertise to state that I do not consider it to be of an acceptable scientific standard, for reasons outlined above.

# Response

**Published:** 2024-11-25

**Authors:** Kunal Anand

**Affiliations:** School of Computer Engineering, Kalinga Institute of Industrial Technology, Bhubaneswar, Odisha, India

**Abstract: The abstract should offer a succinct overview of the research paper, covering the research problem, objectives, methodology, key findings, and conclusions. It must be clear and informative, providing readers with an understanding of the study's importance. You should precisely highlight the main contributions, including the datasets utilized and the accuracy achieved by your proposed system.**

**Response:** We are deeply appreciative of the esteemed reviewer's perceptive comments on our work. In this context, we would like to submit that, as suggested by the esteemed reviewer, the abstract has been reworked in the revised manuscript.

**Introduction: The introduction section should conclude with a brief outline of the structure for the remainder of the paper.**

**Response:** We sincerely appreciate the insightful feedback on our efforts provided by the excellent reviewer. In light of this, we would like to state that the last paragraph of the introduction section outlines the structure for the remainder of the paper.  

 .

**Literature Review: A distinct section dedicated to the Literature Review should be included in the paper in the form of a table.**

**Response:** The excellent reviewer provided us with insightful feedback of our work, which we much appreciate. This is a really smart idea from the respected critic. As desired by the respected reviewer, we would like to submit that the literature review has been appended as a table at the conclusion of the related work section.

**Results: The results section should clearly and systematically present the study's findings. It should provide detailed information, including a thorough comparison with other related techniques. You also have to explore other measures as well in the comparison other techniques.**

**Response:** We are deeply appreciative of the distinguished reviewer's insightful critique of our work. It is a very wise suggestion from the renowned critic. We have incorporated in the amended text the notable distinctions between the suggested approach and the other related FS techniques employed in this work, as suggested by the excellent reviewer. Furthermore, a distinct section titled "Threats to validity" in the updated manuscript addresses the ramifications of the suggested FSCOA strategy. In addition to investigating additional measurements, our goal for the future is to broaden the scope of the suggested work by comparing it with more FS techniques.

**Conclusion: This section should offer a comprehensive summary of the research and include a detailed discussion of future research directions.**

**Response:** We greatly appreciate the insightful criticism of our work provided by the esteemed reviewer. The renowned reviewer has offered a really astute suggestion. In light of this, we would like to submit that the conclusion section has been altered in the revised manuscript in accordance with the distinguished reviewer's suggestions.

**English: The paper should be reviewed to ensure the English language is free of grammatical errors.**

**Response:** To make sure the English is correct, the paper has been carefully examined and modified accordingly.
